# Klf9 promotes the repair of myocardial infarction by regulating macrophage recruitment and polarization

**DOI:** 10.1172/jci.insight.187072

**Published:** 2025-04-08

**Authors:** Sheng Xu, Hao Li, Jun Han, Yawei Xu, Niannian Li, Wenliang Che, Feng Liu, Wenhui Yue

**Affiliations:** 1 Department of Cardiology, Shanghai Tenth People’s Hospital, Tongji University School of Medicine, Shanghai, China.; 2 Department of Otolaryngology Head and Neck Surgery, Shanghai Key Laboratory of Sleep Disordered Breathing, Shanghai Jiao Tong University School of Medicine Affiliated Sixth People’s Hospital, Shanghai, China.

**Keywords:** Cardiology, Inflammation, Cardiovascular disease, Fibrosis, Macrophages

## Abstract

The inflammatory response after myocardial infarction (MI) is a precisely regulated process that greatly affects subsequent wound healing and remodeling. However, understanding about the process is still limited. Macrophages are critically involved in inflammation resolution after MI. Krüppel-like factor 9 (*Klf9*) is a C2H2 zinc finger–containing transcription factor that has been implicated in glucocorticoid regulation of macrophages. However, the contribution of *Klf9* to macrophage phenotype and function in the context of MI remains unclear. Our study revealed that KLF9 deficiency resulted in higher mortality and cardiac rupture rate, as well as a considerable exacerbation in cardiac function. Single-cell RNA sequencing and flow cytometry analyses revealed that, compared with WT mice, *Klf9^–/–^* mice displayed excessive neutrophil infiltration, insufficient macrophage infiltration, and a reduced proportion of monocyte-derived CD206^+^ macrophages after MI. Moreover, the expression of IFN-γ/STAT1 pathway genes in *Klf9^–/–^* cardiac macrophages was dysregulated, characterized by insufficient expression at 1 day post-MI and excessive expression at day 3 post-MI. Mechanistically, *Klf9* directly binds to the promoters of *Stat1* gene, regulating its transcription. Overall, these findings indicate that *Klf9* beneficially influences wound healing after MI by modulating macrophage recruitment and differentiation by regulating the IFN-γ/STAT1 signaling pathway.

## Abstract 2

*Klf9* beneficially influence wound healing after MI through modulating macrophage recruitment and differentiation by regulating the IFN-γ-STAT1 signal pathway

## Graphical abstract

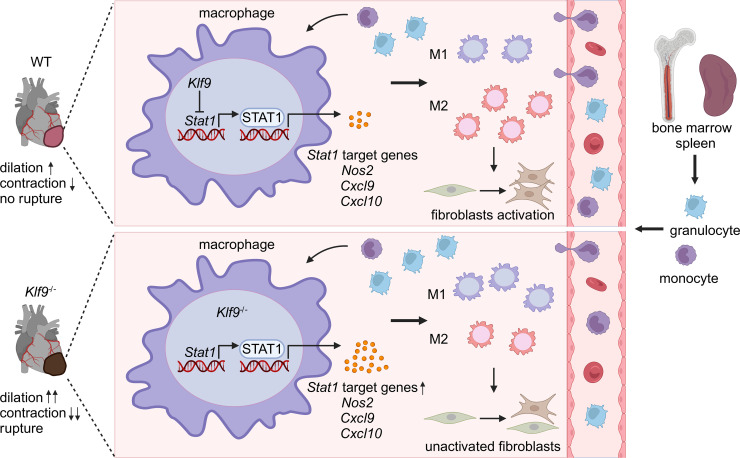

## Introduction

Cardiovascular disease (CVD) is the leading cause of death worldwide (1). Myocardial infarction (MI), resulting from thrombosis or vascular occlusion, is one of the most common causes of CVD death (2). Despite thrombolysis and reperfusion strategies (3, 4), therapeutic manipulation of the subsequent repair process has proven challenging and elusive (5, 6). Cardiac repair post-MI is initiated by an inflammatory phase that involves immune cell infiltration and clearance of damaged tissues, followed by a reparative phase with inflammation resolution, myofibroblast proliferation, angiogenesis, and scar formation (7). A proper balance between these 2 phases is critical for optimal repair post-MI. Therefore, elucidating the regulatory mechanisms of inflammation during cardiac repair is necessary for pro-repair therapies.

Monocytes/macrophages in MI tissue exhibit phasic functional heterogeneity and contribute essentially to wound healing post-MI (8). Ly6C^hi^ monocytes and M1 macrophages with a pro-inflammatory phenotype rapidly aggregate, as well as highly express proteases and inflammatory factors, thus facilitating removal of debris early (9). Subsequently, Ly6C^lo^ monocytes and reparative macrophages gradually replace pro-inflammatory macrophages and secrete antiinflammatory, pro-fibrotic, and pro-angiogenic factors (9, 10). Previous studies have shown that ablation of macrophages or overinhibiting inflammatory signals to suppress excessive inflammatory responses may lead to poor healing, unstable scar formation, and even heart rupture (11, 12). Therefore, appropriate and timely phenotypic transformation of macrophages could restrict inflammation and facilitate optimal wound healing post-MI (13).

Krüppel-like factor 9 (*Klf9*) is a C2H2 zinc finger–containing transcription factor that is conserved in mammals and is regulated by a variety of hormones, including progesterone, thyroid hormone, and glucocorticoids (14–17). In addition, *Klf9* is involved in the regulation of the immune system. Overexpression of KLF9 can regulate B cell proliferation and convert memory B cells into cells with a “naive” B cell phenotype, possibly through interaction with the NF-κB pathway downstream of CD40 and B cell receptor signaling (18). In macrophages, overexpression of *Klf9* inhibits the expression of some M1 and M2a marker genes, and the reduced secretion of these signaling molecules inhibits STAT3 signaling in adipocytes, thereby participating in glucocorticoid-induced obesity (19).

*Klf9* is positively regulated by the glucocorticoid receptor, and glucocorticoids are known to promote phagocytosis of monocytes/macrophages and activate antiinflammatory gene programs, inducing macrophage differentiation into the M2 type (20, 21). It is still unclear whether *Klf9* is also involved in this process and whether it plays a role in the inflammation and repair of MI. In our study, we found that *Klf9* knockout markedly inhibited the repair of MI in the early stage. This is because *Klf9* deficiency weakened the chemotaxis and enhanced the inflammatory response of macrophages, which led to insufficient infiltration of reparative macrophages during the repair phase and slowing of the initiation of fibrosis. Mechanistically, *Klf9* regulates macrophage recruitment and polarization mainly through temporal regulation of the STAT1 pathway.

## Results

### KLF9 deficiency inhibits post-MI repair and leads to increased mortality.

**Figure 1:**
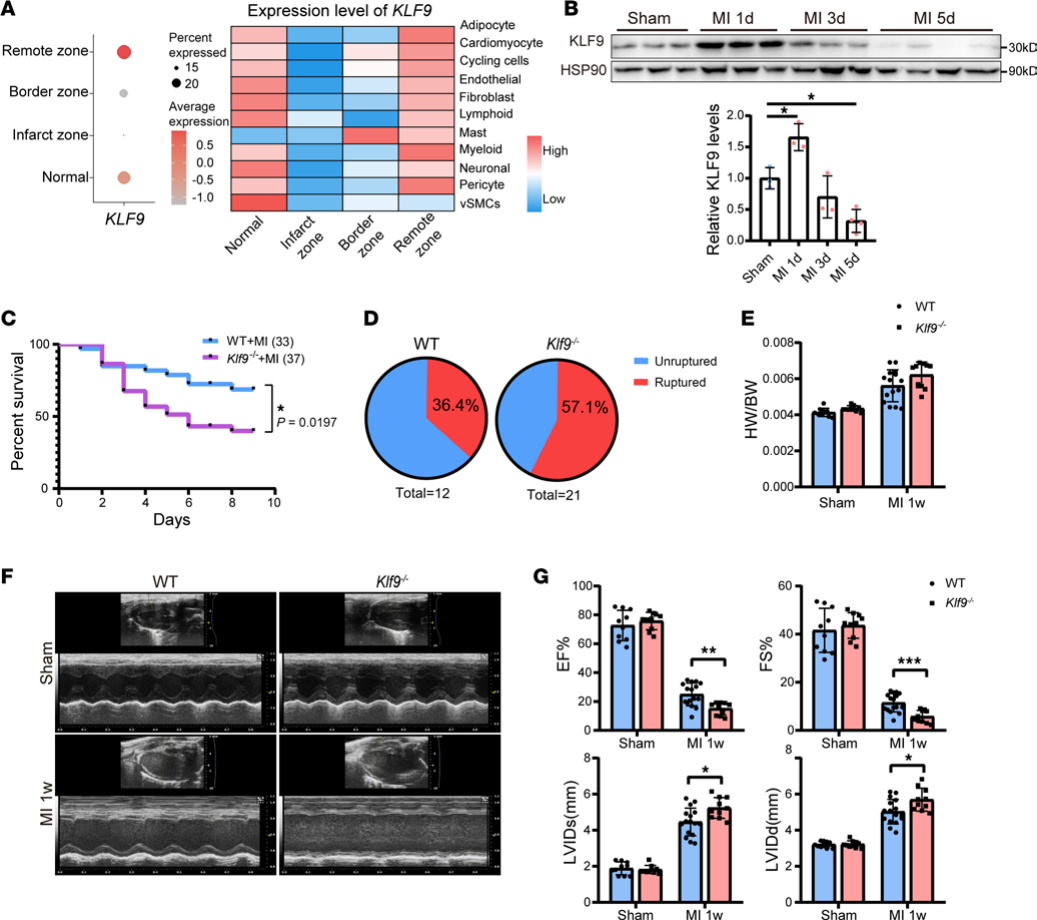
KLF9 deficiency leads to increased mortality. (**A**) Expression of *KLF9* in normal heart and different zones of MI heart in human single-cell RNA-Seq data (22) (left) and the expression of *KLF9* in different cell types (right). (**B**) KLF9 protein levels in the injured zone (infarct zone + border zone) of hearts (*n* = 3–4). (**C**) Survival curve (log-rank [Mantel-Cox] test) of WT and *Klf9^–/–^* mice subjected to MI (WT *n* = 33, *Klf9^–/–^* *n* = 37). (**D**) The percentage of ruptured and nonruptured hearts in dead mice in **C** (WT *n* = 12, *Klf9^–/–^* *n* = 21). (**E**) Ratio of heart weight (HW) to body weight (BW) in WT and *Klf9^–/–^* groups 1 week post-MI (*n* = 10–15). (**F** and **G**) Representative M-mode images of WT and *Klf9^–/–^* mice 1 week post-MI and the measurement of left ventricular ejection fraction (EF), fractional shortening (FS), left ventricular internal diameter at end-systole (LVIDs), and left ventricular internal diameter at end-diastole (LVIDd) (*n* = 10–16). Each point represents a mouse sample, and all data are expressed as means ± SEM. Unpaired 2-tailed Student’s *t* test (**E** and **G**), 1-way ANOVA (**B**) was used for statistical analyses. *P < 0.05, **P < 0.01, ***P < 0.001.

To explore whether KLF9 is involved in the pathological process of MI, we analyzed the published single-cell sequencing data of human MI (22), which included normal heart and heart tissues from different regions during the acute phase of MI (within 15 days). The results showed that the proportion of cells expressing *KLF9* decreased in the infarct and border zone after MI (Figure 1A), and the expression level of *KLF9* decreased in various cell types (Figure 1A), suggesting that *KLF9* is involved in MI, especially in the infarct zone. Subsequently, we constructed an MI model in C57BL/6J mice and monitored the KLF9 expression in the injured zone (infarct zone + border zone). The results showed that KLF9 increased significantly 1 day post-MI and then gradually declined, and KLF9 significantly decreased 5 days post-MI (Figure 1B), indicating that the effect of KLF9 on MI may be time dependent. To further characterize the functional role of KLF9 in MI, we constructed *Klf9*-knockout (*Klf9^–/–^*) mice and constructed an MI model in WT and *Klf9^–/–^* mice. The results showed that the survival of *Klf9^–/–^* mice was significantly decreased (Figure 1C), and among the dead mice, the proportion of heart rupture in *Klf9^–/–^* mice was higher compared with the WT controls (Figure 1D). Echocardiography results showed that *Klf9^–/–^* mice had worse cardiac function and more severe ventricular dilation (Figure 1, F and G).

**Figure 2:**
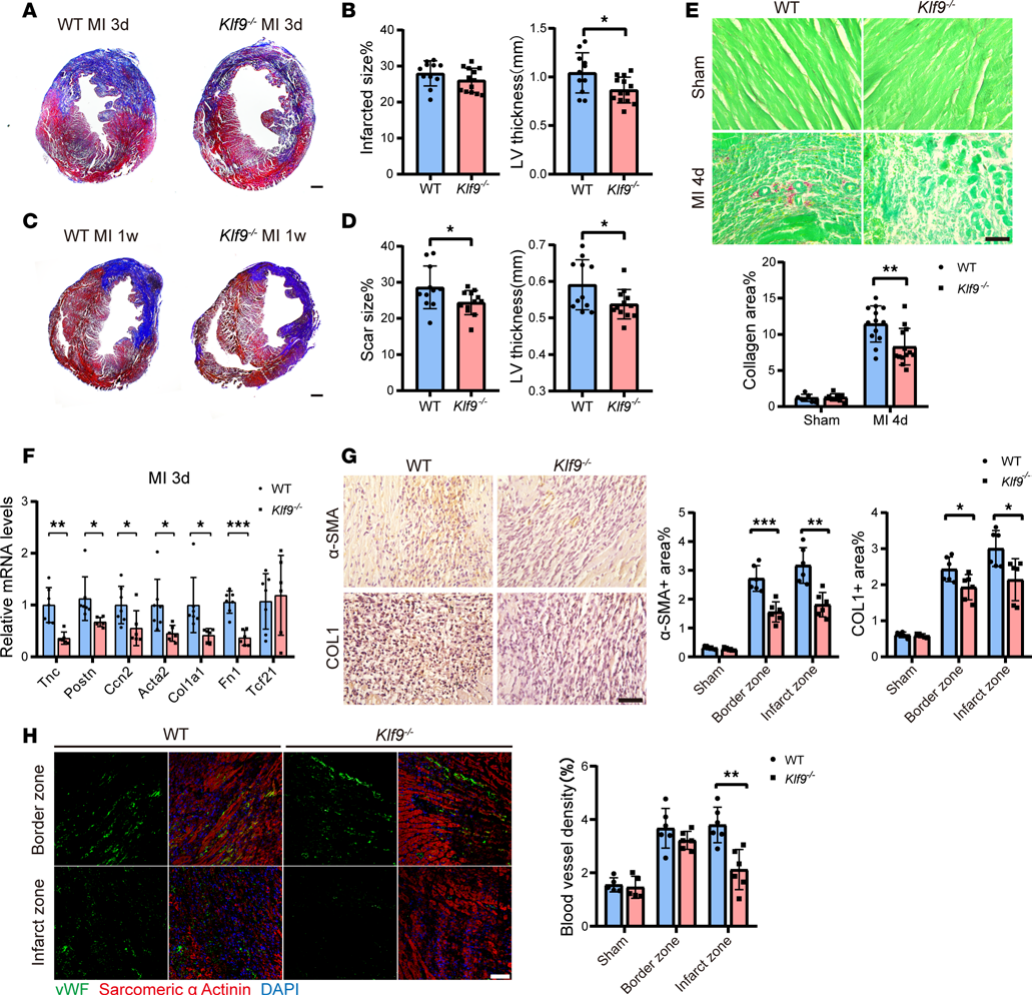
KLF9 is involved in the early repair process post-MI. (**A**–**D**) Representative photographs of Masson’s trichrome staining and quantitative data of infarcted/scar size% and left ventricular (LV) thickness at 3 days (**A** and **B**) and 1 week (**C** and **D**) post-MI (*n* = 11–13, scale bar = 0.5 mm). (**E**) Representative images of Sirius red staining and quantification of collagen area% in cardiac tissue 4 days post-MI (sham *n* = 8, MI4d *n* = 13, scale bars = 50 μm). (**F**) Real-time quantitative PCR (RT-qPCR) of myofibroblast activation marker genes was performed in cardiac tissues from the injured zone 3 days post-MI. The gene expression was analyzed using the ΔΔCt method (WT *n* = 7, *Klf9^–/–^* *n* = 6). (**G**) Representative images and quantification of α-SMA and collagen 1 (COL1) IHC staining in heart tissues 4 days post-MI (Sham *n* = 5, MI4d *n* = 6, Scale bars = 50 μm). (**H**) Representative immunofluorescence staining and quantification of vWF^+^ endothelial cells in the hearts 4 days post-MI (*n* = 5–6, scale bars = 50 μm). Each point represents a mouse sample, and all data are expressed as means ± SEM. Unpaired 2-tailed Student’s *t* test (**B** and **D**–**H**) was used for statistical analyses. *P < 0.05, **P < 0.01, ***P < 0.001.

However, there were no significant differences in HW/BW ratio (Figure 1E) and cross-sectional area of cardiomyocytes (Supplemental Figure 2A; supplemental material available online with this article; https://doi.org/10.1172/jci.insight.187072DS1) 1 week post-MI. Masson’s staining showed that by 3 days post-MI, there was no significant difference in the infarct zone between WT and *Klf9^–/–^* mice (the blue area indicates fibrotic tissue and necrotic cells), but the ventricular wall of the infarcted zone of *Klf9^–/–^* mice was thinner (Figure 2, A and B); 1-week post-MI, the scar size of *Klf9^–/–^* heart was smaller (the blue area indicates fibrotic tissue), and the ventricular wall of the infarcted zone was also thinner (Figure 2, C and D).

In addition, we observed the long-term outcomes post-MI. At 4 weeks post-MI, the cardiac function of *Klf9^–/–^* mice was further reduced (Supplemental Figure 1, A–C), and the pathological results were similar to those from 1 week post-MI (Supplemental Figure 1, D and F). However, there was no significant difference in scar area (Supplemental Figure 1E), and *Klf9^–/–^* mice had larger HW/BW ratio, suggesting the development of cardiac hypertrophy (Supplemental Figure 1G). Taken together, these results suggested that expression of *Klf9* is protective against MI, and knockout leads to increased mortality, greater likelihood of heart rupture, worsened cardiac function, and aggravated ventricular remodeling. These phenotypes were not caused by enlargement of infarct area in *Klf9^–/–^* mice but were more likely due to abnormal repair.

At 3–5 days after MI in mice, fibroblasts enter a proliferation phase characterized by activation, differentiation into myofibroblasts, and expression of high concentrations of α–smooth muscle actin (α-SMA), which confers high contractility (23). To investigate whether *Klf9* regulates the initiation of fibrosis post-MI, we examined the marker genes of activated fibroblasts in the injured zone. The mRNA levels of *Postn*, *Acta2*, *Col1a1*, and *Fn1* were significantly decreased in *Klf9^–/–^* heart tissue 3 days post-MI (Figure 2F), and there were no significant differences in sham groups (Supplemental Figure 1H), suggesting that *Klf9* deficiency reduces the activation of fibroblasts post-MI. Additionally, collagen deposition in the injured zone of *Klf9^–/–^* mice was reduced on day 4 post-MI (Figure 2E), and the expression of α-SMA and COL1 proteins was also decreased (Figure 2G). During the second to fourth days post-MI, the capillary network in the border zone of mice began to expand, and a large number of branches and blood vessels grew toward the infarct center (24). We used vWF as a marker of endothelial cells and observed that the microvascular density in the infarct zone of *Klf9^–/–^* mice was significantly lower than that of WT mice 4 days post-MI, while no significant difference was found in the border zone (Figure 2H). These results indicated that *Klf9* contributes to the initiation of repair post-MI, which may be why *Klf9^–/–^* mice are more prone to cardiac rupture.

### Single-cell RNA sequencing reveals abnormal proportion and functions of Klf9^–/–^ macrophages.

Given that *Klf9* is widely expressed in various cells, and the downstream target of KLF9, peroxisome proliferator–activated receptor γ coactivator 1-α (PGC-1α), plays an important role in cardiomyocytes (17, 25, 26), we cannot exclude the potential role of *Klf9* in cardiomyocyte death post-MI. We found there was no significant difference in myocardial injury markers, such as plasma cardiac troponin I (cTnI) and lactate dehydrogenase (LDH), between WT and *Klf9^–/–^* mice 3 days post-MI (Supplemental Figure 2, B and C). These results suggest that KLF9 deficiency does not increase early cardiomyocyte death post-MI. Moreover, we isolated and cultured rat neonatal cardiomyocytes; treated them with oxygen deprivation, glucose-serum deprivation (GD), and oxygen-glucose deprivation to simulate MI conditions; and found that KLF9 was upregulated during GD and oxygen-glucose deprivation, but there was no significant change during oxygen deprivation (Supplemental Figure 2D), indicating that KLF9 responds to glucose deficiency rather than hypoxia in cardiomyocytes. We also isolated cardiomyocytes of neonatal WT and *Klf9*^–/–^ mice and observed lower autophagy levels in *Klf9^–/–^* cardiomyocytes compared with WT controls, whether under normal conditions or GD treatment (Supplemental Figure 2E), which may be due to the role of KLF9 in mitophagy (26). However, KLF9 deficiency did not affect survival of cardiomyocytes after glucose deprivation (Supplemental Figure 2F). These results indicated that KLF9 may be involved in glucose metabolism and autophagy in cardiomyocytes but has no obvious protective effect on GD-induced cell death. The specific role of *Klf9* in cardiomyocytes may need to be further revealed in other models.

**Figure 3:**
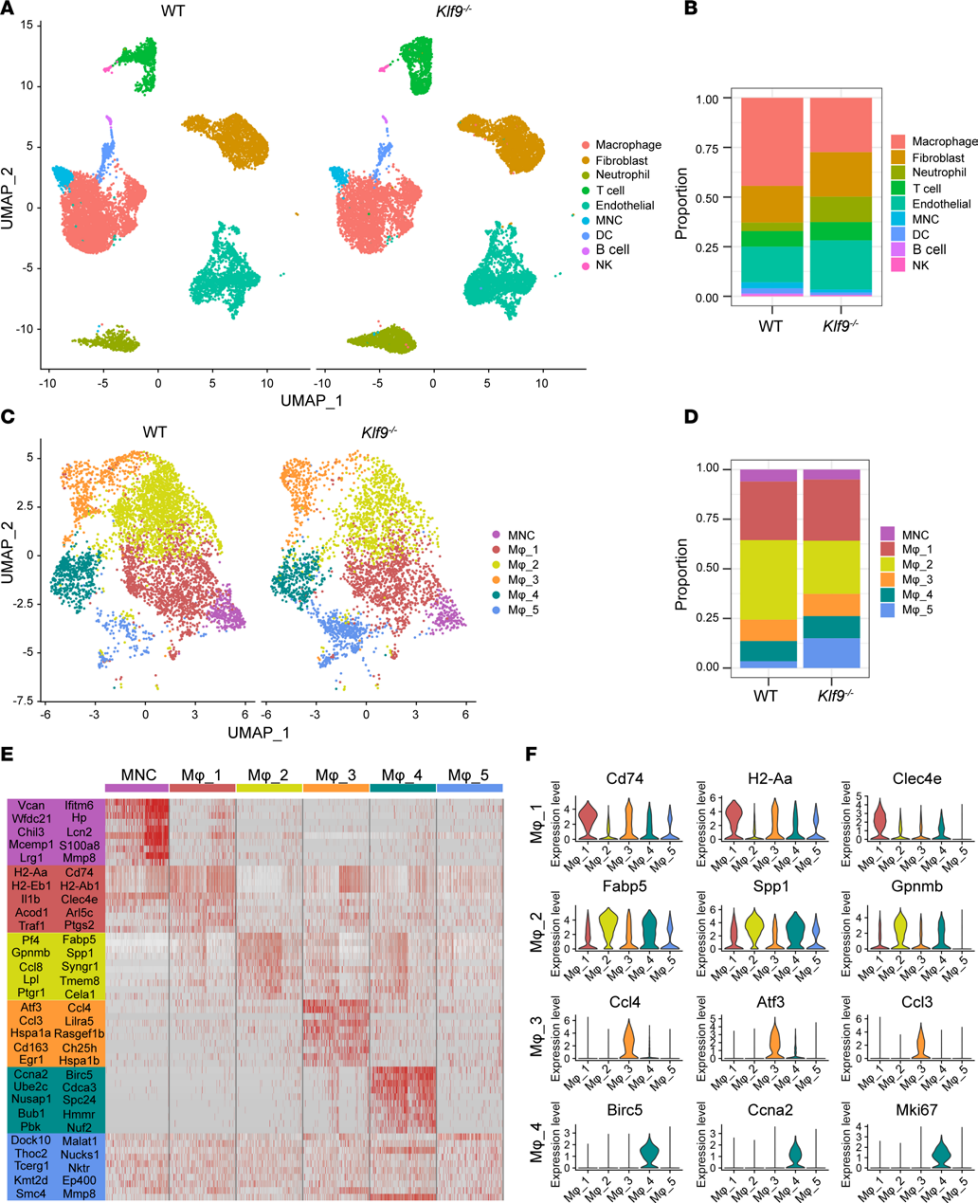
scRNA-Seq reveals the effect of KLF9 on macrophages post-MI. (**A**) Uniform manifold approximation and projection (UMAP) of 9 noncardiomyocyte clusters identified via scRNA-Seq analysis of cardiac tissue 3 days post-MI. (**B**) The proportion of cell clusters in WT and *Klf9^–/–^* groups. (**C**) UMAP of monocyte/macrophage clusters separated from **A**. UMAP shows 5,024 WT cells and 3,097 *Klf9^–/–^* cells. (**D**) The proportion of each subcluster in the monocytes/macrophages in the WT and *Klf9^–/–^* groups. (**E**) Expression heatmap of marker genes in different monocyte/macrophage subclusters. (**F**) Violin plots of some marker genes in macrophage subsets 1–4 (Mφ_1-4).

Given the above results, we isolated noncardiomyocytes from WT and *Klf9^–/–^* mice 3 days post-MI and sent them for single-cell RNA sequencing (scRNA-Seq), with each group of samples consisting of cells from 2 mice. After quality control filtering, we identified 23,296 unique cells, including 10,680 cells from WT samples and 12,616 cells from *Klf9^–/–^* samples. Based on the characteristic gene expression profiles of cells, we separated 9 cell clusters (Figure 3A). By counting the proportions of each cell type, we found that the proportion of macrophages in *Klf9*^–/–^ mice was markedly decreased, accompanied by a higher proportion of neutrophils, while other cell types showed no marked difference between the 2 groups (Figure 3B). Given the low expression of *Klf9* in neutrophils in our scRNA-Seq data, we focused on macrophages.

Monocytes/macrophages were then sent for further clustering and subdivided into 1 monocyte (MNC) subset and 5 macrophage (Mφ) subsets (Figure 3C), with the Mφ_2 subset exhibiting the largest difference in proportion between the WT and *Klf9^–/–^* groups (Figure 3D). The heatmap of the top 10 marker genes’ expression of each macrophage subset showed the rationality of the clustering (Figure 3E). By analyzing the expression of marker genes, we further defined the functional characteristics of each subset (Figure 3F). Mφ_1 was primarily differentiated from monocytes and expressed high levels of antigen presentation–related genes (*H2-Aa*, *H2-Ab1*, and *Cd74*), as well as immune response–related genes (*Clec4e*, *Il1b*, and *Ptgs2*). Mφ_2, which highly expressed *Fabp5*, *Spp1*, and *Gpnmb*, was previously identified as a pro-fibrosis Mφ subset in human liver and lung (27). Mφ_2 was markedly reduced in *Klf9^–/–^* mice, which may be related to the reduction of fibrosis observed in *Klf9^–/–^* mice post-MI. Mφ_3 was defined as a pro-chemotaxis Mφ subset owing to the high expression of chemotaxis-related genes, including *Ccl4*, *Atf3*, *Ccl3*, and so on. Moreover, Mφ_4 consisted of cardiac resident macrophages with proliferative ability, expressing high levels of cell cycle–related genes, including *Birc5*, *Ccna2*, and *Mki67*, and low levels of *Ccr2*. Although important in MI (28), the Mφ_4 subset exhibited no obvious difference between WT and *Klf9*^–/–^ groups. Finally, Mφ_5 was a group of mature macrophages, which highly expressed the pro-inflammatory gene *Malat1* (29) and RNA metabolism regulation–related genes, including *Ep400*, *Thoc2*, and *Tcerg1*.

**Figure 4:**
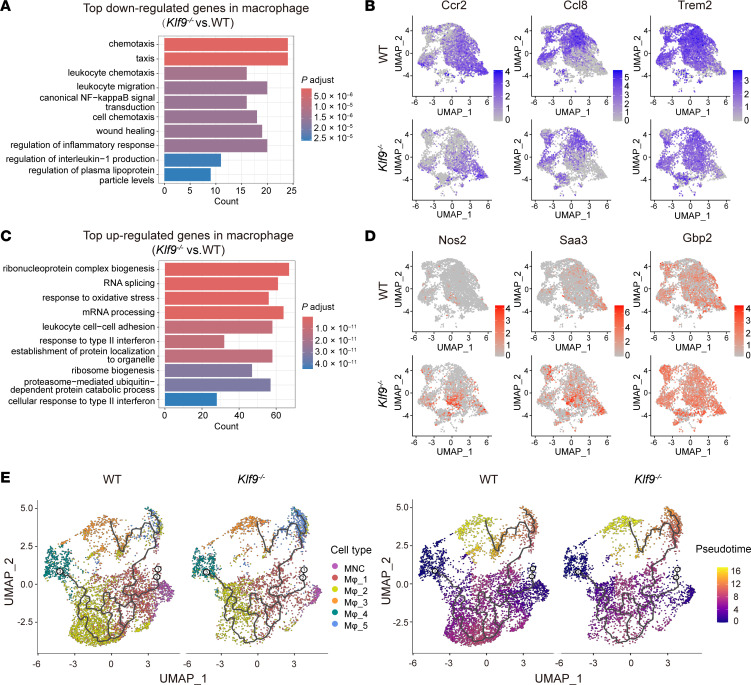
KLF9-deficient macrophages have reduced chemotaxis but increased inflammation. Enrichment GO analysis of the most significantly downregulated genes (**A**) and upregulated genes (**C**) in macrophages (*Klf9^–/–^* vs. WT). Differential gene: log2(FC) < –0.5 or > 0.5, GO domains: Biological Process. (**B** and **D**) UMAP visualization of differential expression of some chemotaxis pathway genes (**B**) and inflammatory pathway genes (**D**). (**E**) Pseudotime trajectory analysis in monocyte/macrophage subsets; cells were colored by pseudotime or subcluster labels.

Functional enrichment of Gene Ontology (GO) analysis of differentially expressed genes (DEGs) in monocytes/macrophages revealed that compared with WT controls, *Klf9^–/–^* macrophages had the most significant downregulation in chemotaxis-related pathways (Figure 4A), with the representative genes including *Ccr2*, *Ccl8*, and *Trem2* (Figure 4B). Conversely, most of the genes upregulated in the *Klf9^–/–^* group were enriched in pathways related to oxidative stress, cell adhesion, and interferon (Figure 4C), with the representative genes including *Nos2*, *Saa3*, and *Gbp2* (Figure 4D). These results indicated that *Klf9* deficiency impairs macrophage chemotaxis and exacerbates macrophage inflammation post-MI. Pseudotime trajectory analysis of monocytes/macrophages revealed that macrophages had 2 main sources: monocytes and proliferative resident macrophages (Figure 4E). UMAP plotting results suggested that the macrophage subpopulations (Mφ_1 and part of Mφ_2) derived from monocytes were markedly reduced in the *Klf9^–/–^* group (Figure 4E). Therefore, we reasonably speculated that the lack of monocyte-derived macrophage chemotaxis was the primary cause of the macrophage reduction post-MI in *Klf9^–/–^* mice, rather than the absence of resident macrophage proliferation.

Fibroblasts, the main executors of fibrosis, were analyzed for the differences between WT and *Klf9^–/–^* groups. Clustering and pseudotime trajectory analysis delineated 3 subsets of fibroblasts, with developmental trajectories from FB_3 to FB_1 (Supplemental Figure 3A). We analyzed the marker genes and developmental trajectories of each subset: FB_1 is the activated myofibroblast subset, FB_3 is the progenitor cells of myofibroblasts, and FB_2 is a transitional subset between the 2 subsets (Supplemental Figure 3, A and B). The proportions of the 3 subsets in the 2 groups showed that the proportion of myofibroblasts in the *Klf9^–/–^* group was markedly decreased compared with that in the WT group, and most of the cells in the *Klf9^–/–^* group were still in an inactivated or transitional state (Supplemental Figure 3C). Therefore, the expression levels of many activation-related genes in *Klf9*^–/–^ fibroblasts were lower (Supplemental Figure 3D). The most significantly downregulated genes in the *Klf9^–/–^* group compared with the WT group also showed enrichment in the extracellular matrix pathway (Supplemental Figure 3E). To investigate whether fibroblast activation is different between *Klf9^–/–^* and WT groups, we isolated mouse neonatal cardiac fibroblasts and seeded them at low density (5 cells per mm^2^). Since fibroblasts will spontaneously activate when cultured in high-glucose DMEM containing 10% FBS and standard plastic plates (30), we detected myofibroblast markers 1 week after culture and found there was no significant difference in the 2 groups of genes except for *Sfrp2* expression (Supplemental Figure 3F).

Taken together, these results suggested that *Klf9* deficiency led to impaired number and function of macrophages 3 days post-MI and further inhibited fibroblasts’ activation, which may eventually lead to slow initiation of repair post-MI.

### KLF9 regulates macrophage chemotaxis and polarization in a temporal manner.

**Figure 5:**
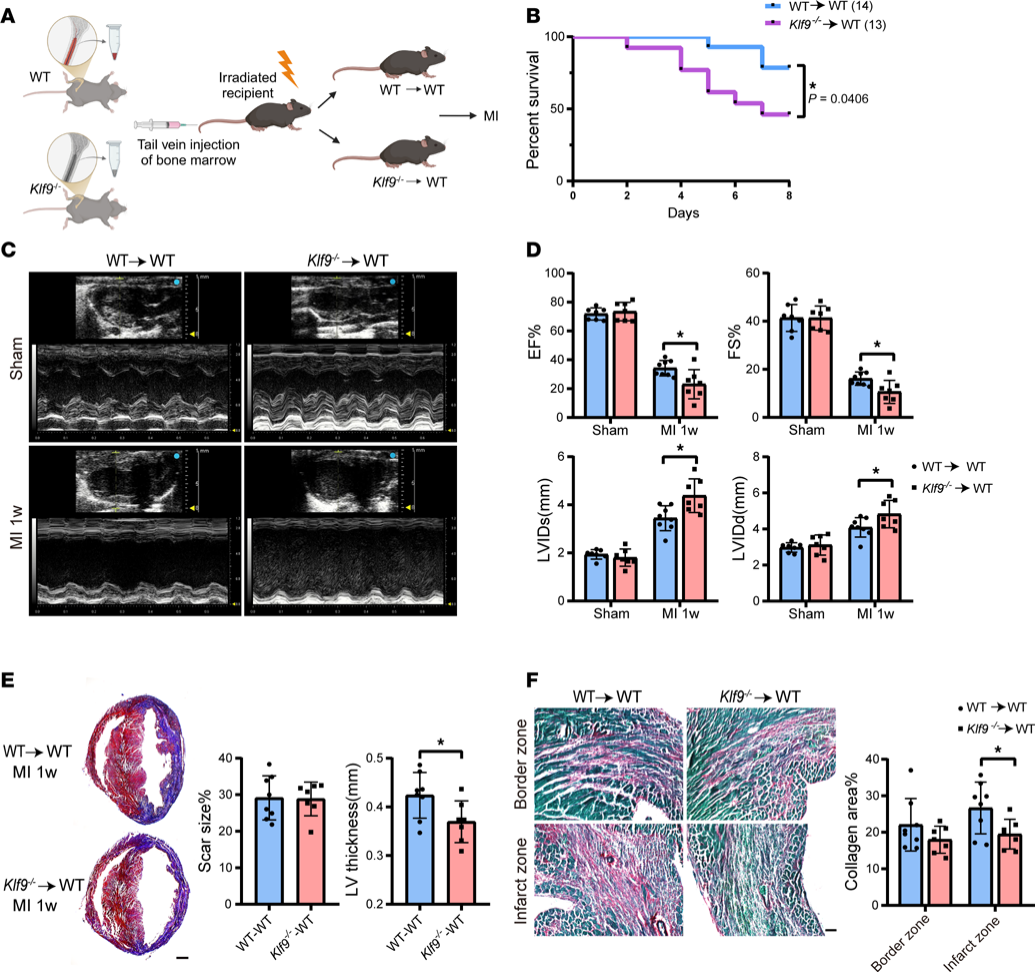
KLF9-deficient bone marrow–derived myeloid cells increased mortality post-MI. (**A**) Schematic diagram of the strategy of the bone marrow transplantation (BMT) experiment. (**B**) Survival curve (log-rank [Mantel-Cox] test) of WT → WT mice (*n* = 14) and *Klf9^–/–^*→ WT mice (*n* = 13) subjected to MI. (**C** and **D**) Representative M-mode images of WT → WT and *Klf9^–/–^*→ WT mice 1 week post-MI and the measurement of EF%, FS%, LVIDs, and LVIDd (*n* = 7–8). (**E**) Representative photographs of Masson’s trichrome staining and quantitative data of scar size% and LV thickness 1 week post-MI (*n* = 7–8, scale bar = 0.5 mm). (**F**) Representative images of Sirius red staining and quantification of collagen area% in cardiac tissue 1 week post-MI (*n* = 7–8, scale bars = 50 μm). Each point represents a mouse sample, and unpaired 2-tailed Student’s *t* test (**D**–**F**) was used for statistical analyses. *P < 0.05.

To verify that KLF9 affects the healing of MI by regulating bone marrow–derived monocytes/macrophages, we performed bone marrow transplantation (BMT) experiments (Figure 5A and Supplemental Figure 3G). Similar to *Klf9^–/–^* mice, mice transplanted with *Klf9^–/–^* bone marrow had increased mortality (Figure 5B) and decreased cardiac function 1 week post-MI (Figure 5, C and D). Correspondingly, KLF9 deficiency in bone marrow cells resulted in thinner ventricular walls, more severe ventricular dilation, and insufficient collagen deposition 1 week post-MI (Figure 5, E and F), though fibrosis and collagen deposition were slowed in bone marrow–transplanted mice compared with normal mice (Figure 2C).

**Figure 6:**
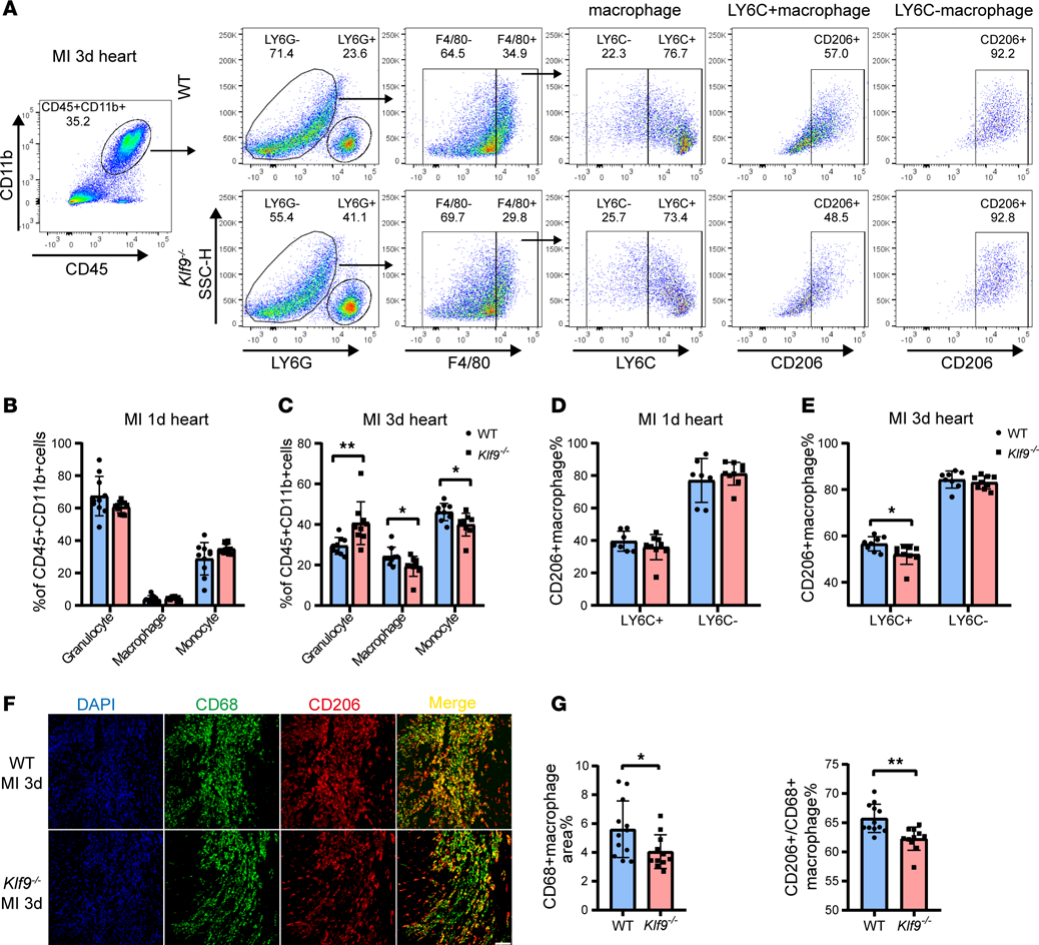
KLF9 regulates the function of monocyte-derived macrophages post-MI. (**A**) Flow cytometry gating strategy and representative flow cytometric analysis of macrophages, granulocytes, and monocytes in WT and *Klf9^–/–^* hearts 3 days post-MI; 20,000 CD45^+^ cells were collected from each mouse sample. (**B** and **C**) Quantification of F4/80^+^LY6G- macrophages, F4/80^–^LY6G^–^ monocytes, and F4/80^–^LY6G^+^ granulocytes in the hearts by flow cytometry at day 1 and day 3 post-MI (*n* = 9–11). (**D** and **E**) Quantification of CD206^+^LY6C^+^ M2 macrophages and CD206^+^LY6C^–^ M2 macrophages in the hearts by flow cytometry at day 1 and day 3 post-MI (*n* = 7–9). (**F** and **G**) Representative immunofluorescence images and quantification of CD68^+^ macrophages and CD68^+^CD206^+^ M2 macrophages 3 days post-MI (WT *n* = 12, *Klf9^–/–^* *n* = 11, scale bars = 100 μm). Each point represents a mouse sample and all data are expressed as means ± SEM. Unpaired 2-tailed Student’s *t* test (**B**–**E** and **G**) was used for statistical analyses. *P < 0.05, **P < 0.01.

Therefore, we focused on the monocytes/macrophages infiltrating the heart post-MI. A few hours to 1 day post-MI, pro-inflammatory monocytes and macrophages are recruited to the injured zone (31). The proportion of M1 macrophages peaks 1 day post-MI, and M2 macrophages become predominant 3 days post-MI (32). To investigate the differences in immune cell infiltration in the early stages of MI, we performed flow cytometric analysis of cardiac immune cells 1 and 3 days post-MI. There was no difference in the proportion of cardiac monocytes and macrophages between groups 1 day post-MI (Supplemental Figure 4A and Figure 6B) or in the number of infiltrating neutrophils (Figure 6B and Supplemental Figure 5, B and C). However, 3 days post-MI, the proportion of monocytes and macrophages infiltrating the *Klf9^–/–^* hearts was significantly decreased, while the proportion of LY6G^+^ granulocytes was relatively increased (Figure 6, A and C). These differences were not due to the ratio of monocytes and granulocytes in the blood (Supplemental Figure 4, E and G). These results suggest that *Klf9* deficiency does not affect the infiltration of macrophages 1 day post-MI but reduces the infiltration of macrophages 3 days post-MI.

We then assessed the polarization of macrophages and found no difference between groups 1 day post-MI (Supplemental Figure 4A and Figure 6D). However, 3 days post-MI, the proportion of CD206^+^ macrophages in the *Klf9^–/–^* group decreased within LY6C^+^ macrophages, with no difference in LY6C^–^ macrophages (Figure 6, A and E). These results suggest that KLF9 deficiency predominantly inhibits M2 polarization of monocyte-derived (LY6C^+^) macrophages but not resident macrophages 3 days post-MI. Immunofluorescence staining of the heart tissues 3 days post-MI also verified reduced macrophage infiltration and CD206^+^ macrophage ratio in *Klf9^–/–^* mice (Figure 6, F and G), with no significant difference observed in the sham group (Supplemental Figure 5A).

These results seem to indicate *Klf9* has no effect on macrophages 1 day post-MI. Notably, there were changes in monocytes and myeloid cells in the blood of the *Klf9^–/–^* group 1 day post-MI, which may be related to the subsequent differences in macrophage infiltration in the heart. The blood *CD45*^+^*CD11b*^+^ myeloid cells in the *Klf9*^–/–^ group were significantly lower than those in the WT group 1 day post-MI (Supplemental Figure 4, B and C), but higher 3 days post-MI (Supplemental Figure 4, E and F), indicating that the blood myeloid cells of *Klf9^–/–^* mice reacted slowly to MI but then compensated.

Interestingly, LY6C expression in cardiac and blood monocytes of *Klf9^–/–^* mice was lower than that of WT mice 1 day post-MI (Supplemental Figure 5, D, E, G, and H), while there was no difference between the 2 groups 3 days post-MI (Supplemental Figure 5, F and I). Previous studies have shown that Ly6C^hi^ monocytes express higher CCR2 than Ly6C^lo^ monocytes and infiltrate more in the MI site (33, 34). However, the difference in LY6C expression in our study is not the difference in the cell populations of LY6C^hi^ and LY6C^lo^. Whether the expression of LY6C is related to the chemotaxis of monocytes requires further investigation.

### KLF9 promotes repair after MI by regulating the macrophage STAT1 signaling pathway.

**Figure 7:**
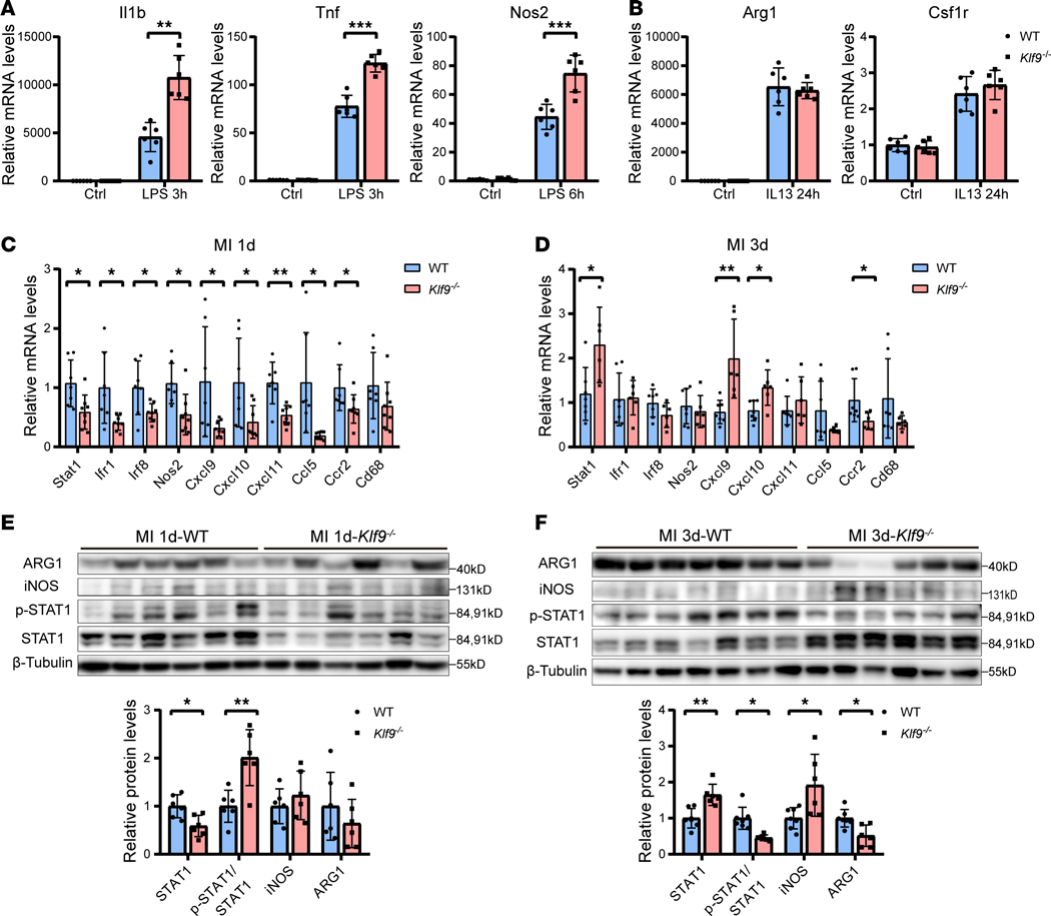
KLF9 regulates STAT1 expression in cardiac tissue post-MI. (**A**) Quantification of inflammation-related mRNAs in BMDMs after LPS treatment (*n* = 6, LPS 50 ng/mL). (**B**) Quantification of macrophage M2 polarization marker genes in BMDMs after IL-13 treatment (*n* = 6, IL-13 10 ng/mL). (**C** and **D**) Relative mRNA expression levels of STAT1 pathway–related genes, *Ccr2*, and *Cd68* in the injured zone of the hearts at day 1 and day 3 post-MI (MI1d *n* = 7–8, MI3d *n* = 6–7). (**E** and **F**) STAT1, phosphorylated STAT1 (p-STAT1), iNOS, and ARG1 expression and quantification in the injured zone day 1 and day 3 post-MI (*n* = 6–7). Each point represents a cell sample (**A** and **B**) or a mouse sample (**C**–**F**), and all data are expressed as means ± SEM. Unpaired 2-tailed Student’s *t* test (**A**–**F**) was used for statistical analyses. *P < 0.05, **P < 0.01, ***P < 0.001.

To explore how *Klf9* affects macrophage polarization, we isolated bone marrow–derived macrophages (BMDMs) from WT and *Klf9^–/–^* mice and stimulated them with LPS or IL-13. Upon LPS treatment, *Klf9^–/–^* BMDMs exhibited significantly higher expression of inflammation-related genes, such as *Il1b*, *Tnf*, and *Nos2*, compared with WT controls (Figure 7A). However, after IL-13 treatment, there was no significant difference of M2 polarization–related genes, such as *Arg1* and *Csf1r*, between the WT and *Klf9^–/–^* BMDMs (Figure 7B). These results indicate that *Klf9* deficiency does not affect M2 polarization of macrophages, but promotes M1 polarization, leading to increased inflammation. We then focused on STAT1, a key transcription factor in macrophage M1 polarization (35). Our scRNA-Seq data revealed *Stat1* and its related *Irf1* and *Irf8* expressions were increased in *Klf9^–/–^* macrophages compared with the WT group 3 days post-MI (Supplemental Figure 6A). By analyzing the mRNA expression in the injured zone of mouse hearts, we found that the expression of *Stat1* and downstream genes in the *Klf9^–/–^* group was significantly decreased compared with the WT group 1 day post-MI, except for the macrophage marker *CD68* (Figure 7C), whereas the expression of these genes was partially decreased in the sham group (Supplemental Figure 6B). However, 3 days post-MI, the expression of *Stat1* and its downstream *Cxcl9* and *Cxcl10* in the *Klf9^–/–^* group increased compared with the WT group (Figure 7D). These results indicates that *Klf9* has a regulatory effect on the STAT1 pathway, which is temporally dependent post-MI. To verify the effect of *Klf9* on STAT1 protein, we also measured the protein expression in the injured zone of the hearts after sham and MI treatment. At 1 day post-MI, the STAT1 level in the *Klf9^–/–^* group was lower (Figure 7E), and it was higher 3 days post-MI than that in the WT group (Figure 7F). In addition, the increase of iNOS and the decrease of ARG1 in the *Klf9^–/–^* heart further verified the reduction of M2 macrophages in this group (Figure 7F). There was no significant difference in STAT1 protein in the sham group (Supplemental Figure 6C).

**Figure 8:**
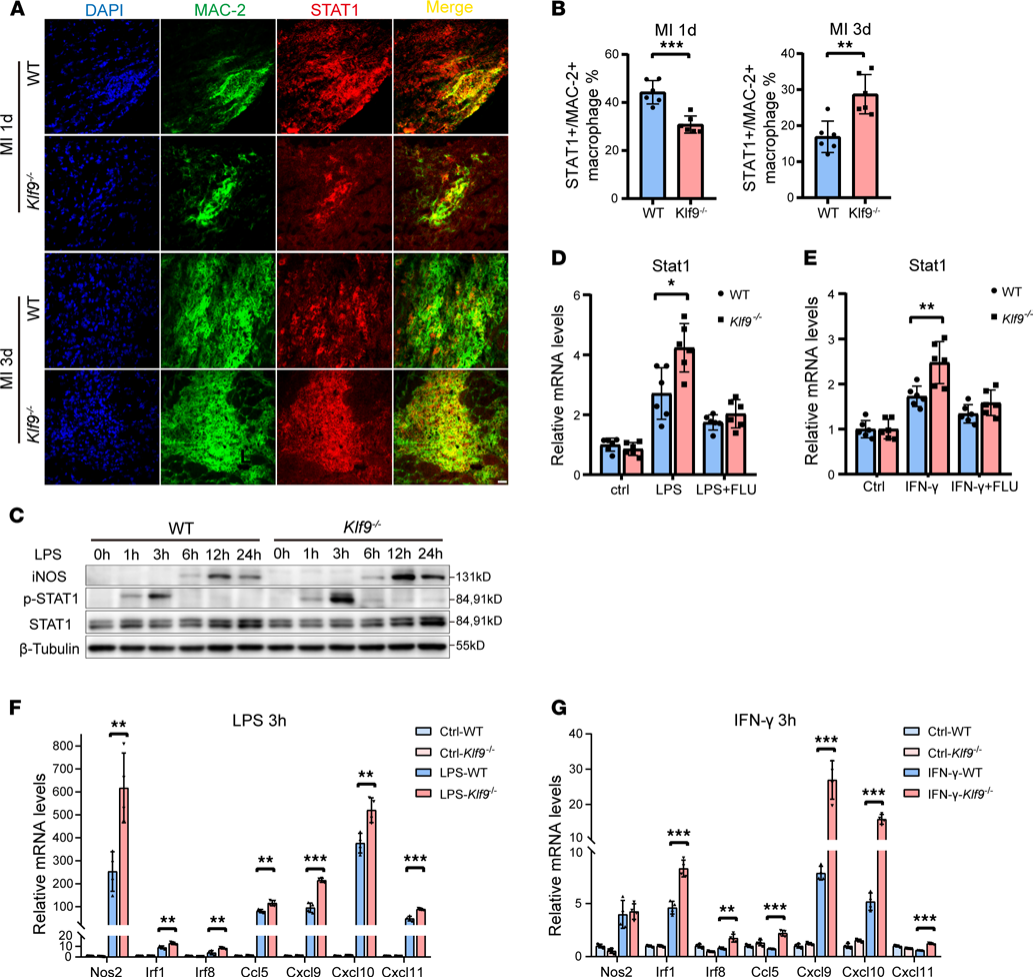
KLF9 regulates STAT1 signaling in macrophages. (**A** and **B**) Immunofluorescence images and quantification of percentage of STAT1^+^MAC-2^+^ macrophages to total macrophages area in the hearts 1 and 3 days post-MI (*n* = 6, scale bars = 20 μm). (**C**) Expression of STAT1, p-STAT1, and iNOS in BMDMs at different time points after LPS treatment. (LPS 50 ng/mL). (**D** and **E**) BMDMs pretreated with DMSO or 50 μM fludarabine for 6 hours, then stimulated with LPS (**D**) or IFN-γ (**E**) for 3 hours, followed by analysis of *Stat1* expression by RT-qPCR. (*n* = 6, LPS 50 ng/mL, IFN-γ 50 ng/mL). (**F** and **G**) Quantification of *Stat1* target gene mRNA expression in BMDMs after 3 hours of LPS (**F**) or IFN-γ (**G**) treatment (*n* = 4, LPS 50 ng/mL, IFN-γ 50 ng/mL). Each point represents a mouse sample (**B**) or a cell sample (**D**–**G**), and all data are expressed as means ± SEM. Unpaired 2-tailed Student’s *t* test (**B** and **D**–**G**) was used for statistical analyses. *P < 0.05, **P < 0.01, ***P < 0.001.

Since STAT1 is widely expressed in various cells, we used MAC-2 as a macrophage marker to detect the expression of STAT1 and found that STAT1 was highly expressed in cardiac macrophages after MI, and the expression of STAT1 in *Klf9*^–/–^ macrophages was lower 1 day post-MI and higher 3 days post-MI compared with the WT group (Figure 8, A and B, and Supplemental Figure 6D). We thus speculated that insufficient STAT1 expression in *Klf9^–/–^* macrophages 1 day post-MI affects the release of multiple downstream chemokines, which may be one of the reasons for insufficient macrophage infiltration, and that high STAT1 expression in *Klf9^–/–^* macrophages 3 days post-MI enhances the inflammatory response of macrophages and inhibits M2 polarization.

### Klf9 can directly bind to the promoter of Stat1 and regulate its transcription.

**Figure 9:**
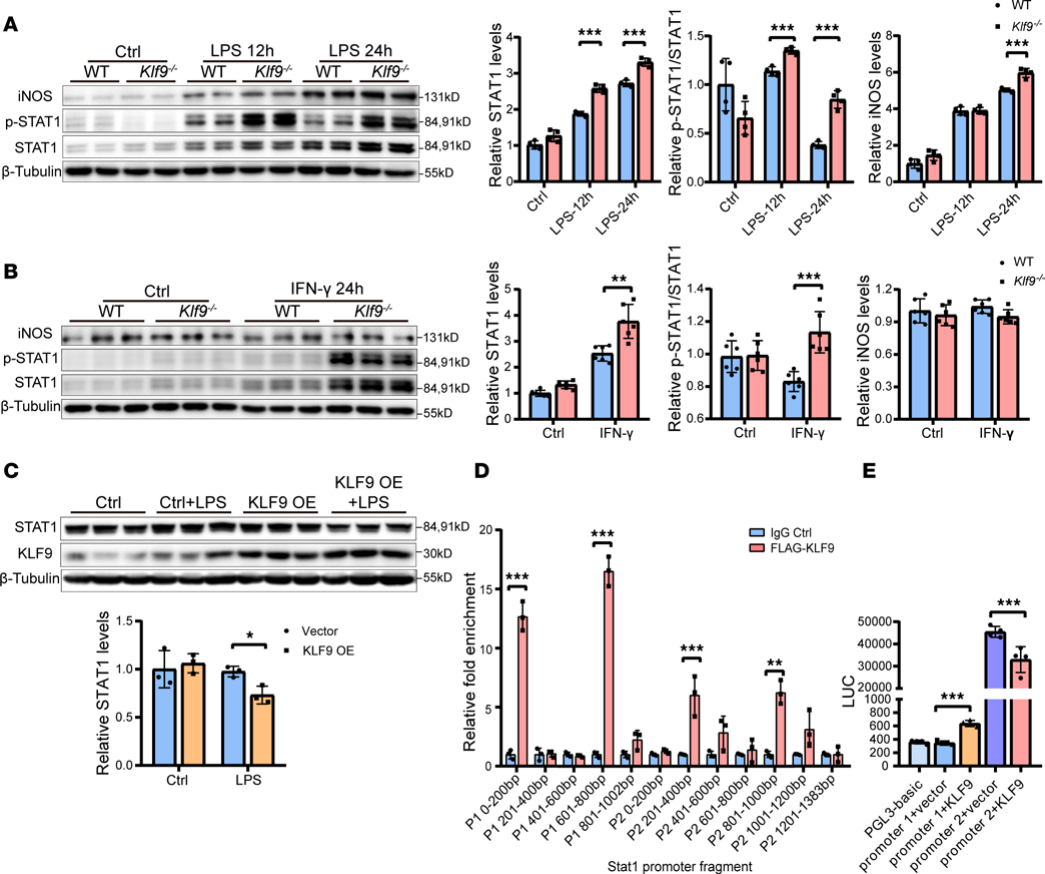
KLF9 regulates macrophage inflammatory response by binding to the *Stat1* promoter. (**A**) STAT1, p-STAT1, and iNOS protein levels and quantification in BMDMs at 12 hours and 24 hours after LPS treatment (*n* = 4, LPS 50 ng/mL). (**B**) STAT1, p-STAT1, and iNOS protein levels and quantification in BMDMs at 24 hours after IFN-γ treatment (*n* = 6, IFN-γ 50 ng/mL). (**C**) STAT1 protein levels and quantification in HeLa cells transfected with pCDNA3.1 vector or pCDNA3.1-KLF9 after 24 hours of LPS treatment (*n* = 3, LPS 100 ng/mL). OE, overexpression. (**D**) ChIP was performed against KLF9 on FLAG-KLF9–overexpressing RAW264.7 cells followed by RT-qPCR with primers specific for *Stat1*’s 2 promoter regions (transcription start site and −6 kb upstream regions) (*n* = 3). (**E**) Relative firefly luciferase (LUC) luminescence in HEK293T cells transiently transfected with pCDNA3.1 vector or pCDNA3.1-KLF9 cotransfected with PGL3 reporter vector (*n* = 4). Each point represents a cell sample, and all data are expressed as means ± SEM. Unpaired 2-tailed Student’s *t* test (**A**–**D**), 1-way ANOVA (**E**) used for statistical analyses. *P < 0.05, **P < 0.01, ***P < 0.001.

To study the regulation of KLF9 on STAT1 in macrophages, we stimulated WT and *Klf9^–/–^* BMDMs with LPS and detected the levels of total STAT1, p-STAT1, and iNOS at different time points (Figure 8C). The phosphorylation of STAT1 peaked at 3 hours after LPS stimulation and was higher in the *Klf9^–/–^* BMDMs than WT controls. STAT1 protein levels began to increase 12–24 hours after LPS stimulation and were higher in the *Klf9^–/–^* BMDMs than WT controls. Correspondingly, similar changes were observed in downstream iNOS levels (Figure 8C). Therefore, we examined the mRNA changes of *Stat1* and its downstream genes at 3 hours after LPS and IFN-γ treatment. After LPS treatment, the expression of *Stat1* and its downstream genes in *Klf9^−/−^* BMDMs increased more rapidly than that in WT controls (Figure 8, D and F). Stimulation of BMDMs with IFN-γ yielded the same results as LPS (Figure 8, E and G). Furthermore, excessive expression of *Stat1* and its downstream genes caused by KLF9 deficiency was abolished by treatment with the STAT1 inhibitor fludarabine (Figure 8, D and E, and Supplemental Figure 6, E and F). The STAT1 protein and p-STAT1 levels in *Klf9^–/–^* BMDMs were also higher than those in the WT group after 12 hours and 24 hours of LPS treatment or IFN-γ treatment (Figure 9, A and B). We then overexpressed human KLF9 in HeLa cells and observed no significant change in STAT1 expression under normal conditions, but after LPS stimulation, KLF9 significantly downregulated the STAT1 levels (Figure 9C). These results indicated that KLF9 can suppress the expression of STAT1 in BMDMs, resulting in a more intense and persistent inflammatory response in *Klf9^–/–^* BMDMs.

To further explore the specific molecular mechanism by which KLF9 regulates STAT1, we investigated a public ChIP-Seq database (Cistrome DB) and found that KLF9 binds to 2 promoter regions of STAT1 in several human cell types (Supplemental Figure 7A). Similarly, we identified 2 promoter regions upstream of the mouse *Stat1* gene using the Ensembl database. We chose 2 promoter regions of mouse (GRCm39) *Stat1*: promoter 1 (chromosome 1: 52152400-52153401) (P1) and promoter 2 (chromosome 1: 52157800-52159182) (P2) for the further investigation. We performed ChIP experiments in RAW264.7 cells overexpressing Flag-KLF9, followed by RT-qPCR and PCR experiments to examine the binding of KLF9 to different positions of P1 and P2. The results showed that KLF9 binds to multiple positions in the *Stat1* promoter (Figure 9D and Supplemental Figure 7B). We applied the firefly fluorescent luciferase reporter assay to analyze the binding of KLF9 to these 2 promoter regions (Figure 9E). Our results showed that P2 had a stronger transcriptional activation ability, while KLF9 exerted transcriptional inhibition on P2 but a transcriptional activation effect on P1. Therefore, we speculated that KLF9 can directly bind to the 2 promoter regions of *Stat1* and regulate its transcription. This regulation of *Stat1* expression subsequently influences its protein levels and phosphorylation status, thereby regulating the expression of pro-inflammatory genes downstream of *Stat1* and affecting the recruitment and polarization of macrophages.

KLF9 regulates the temporal expression of STAT1 post-MI, and the early high expression and subsequent decrease of STAT1 in macrophages underlie their transition from pro-inflammatory to antiinflammatory phenotype. Dysregulation of STAT1 expression will delay the initiation of fibrosis and repair, thereby increasing the risk of cardiac rupture.

## Discussion

Macrophages, especially the recruited monocytes/macrophages, are the crucial effectors and regulators for the inflammation post-MI (36), while tissue-resident *CCR2*^–^ macrophages die locally in the infarct zone (37). Ly6C^hi^*CCR2*^+^ monocytes are recruited to the injured zone, mediated by activation of the monocyte chemoattractant protein-1 (MCP-1)/CCR2 axis (38), and differentiate into pro-inflammatory macrophages to clear injured cardiomyocytes and debris. In our study, the infiltration of *Ccr2*^+^ monocytes/macrophages in the hearts of *Klf9^–/–^* mice post-MI was reduced (Figure 4B and Figure 7, C and D), which could lead to delayed wound healing because necrotic tissue was not cleared in time; thus, granulation tissue and collagen matrix were slowly formed. *CCR2*^+^ macrophages that highly express interferon pathway–related genes (including *Stat1*, *Irf1*, and *Irf7*) are important for the recruitment of monocytes post-MI, primarily by promoting MCP release via a MYD88-dependent mechanism (39). This is consistent with our finding that *Klf9^–/–^* macrophages downregulated STAT1-related pathways 1 day post-MI, followed by reduced recruitment of cardiac monocytes/macrophages. However, despite the reduced number of cardiac macrophages in *Klf9^–/–^* mice, inflammatory pathways were markedly upregulated, accompanied by an increased proportion of neutrophil infiltration after MI. Therefore, we propose that KLF9-deficient macrophages exhibit dysregulated inflammation rather than simply insufficient or excessive inflammation. Although in vitro experiments showed that the alternative pathway of KLF9-deficient macrophages was not impaired (Figure 7B), the number of KLF9-deficient, pro-reparative macrophages was reduced post-MI, due to the complex microenvironment and cytokines. Although pro-inflammatory macrophages secreted excessive inflammatory cytokines during the phase of inflammation resolution, the recruitment of LY6C^lo^ monocytes and their differentiation would be affected.

IFN-γ–mediated JAK/STAT signaling promotes macrophage M1 polarization. IFN-γ receptor activation triggers JAK-mediated phosphorylation and STAT1 dimerization. STAT1 homodimers bind to promoter elements of genes such as NOS2, MHC class II transcription activator, and IL-12 and activate their transcription (40). Studies have shown that *IFN-*γ*^–/–^* mice have significantly reduced cardiac LY6G*^+^* neutrophils and LY6C^hi^ monocytes/macrophages infiltration post-MI because of the absence of IFN-γ/STAT1 signaling and have higher mortality, worse cardiac function, and thinner ventricular walls (41). In our study, *Klf9*^–/–^ mice had a similar phenotype as *IFN-*γ*^–/–^* mice post-MI. The STAT1 signaling pathway in the injured zone of *Klf9^–/–^* mice was significantly downregulated, the proportion of blood myeloid cells was decreased 1 day post-MI, and the number of cardiac monocytes/macrophages decreased 3 days post-MI, all of which were the manifestation of IFN-γ signaling deficiency (Figure 7C). Differently, *Klf9* showed a promotion-following-inhibition pattern in the regulation of IFN-γ/STAT1 signaling in monocytes/macrophages post-MI. Therefore, the lack of *Klf9* resulted in a contradictory state of macrophages in *Klf9^–/–^* mice, in which both insufficient chemotaxis and excessive inflammation were retained, resulting in insufficient reparative macrophages during the resolution phase of inflammation. IFN-γ signaling activation has been shown to inhibit fibrosis in multiple disease models, in part by reducing M2 macrophage differentiation (40). Therefore, M2 macrophage reduction caused by dysregulated IFN-γ/STAT1 signaling is an important reason for insufficient repair in *Klf9^–/–^* mice post-MI. In addition, *Klf9* is a markedly upregulated transcription factor in primary human macrophages after IFN-γ stimulation (42), which also indicates that there is a connection between *Klf9* and the IFN-γ pathway in macrophages.

Recently published research found that *Klf9* mediates the suppression of macrophage M1 and M2a markers (including *Il6*, *Ptgs2*, *Il10*, *Arg1*, and *Chil3*) by glucocorticoids, causing macrophage deactivation (19). However, *Klf9* did not affect the expression and phosphorylation of the transcription factors Stat3 and P65 upstream of the above marker genes (19). The above findings are consistent with our study. We also found that *Klf9* has antiinflammatory effects in macrophages, especially in primary macrophages isolated in vitro. Moreover, *Klf9^–/–^* cardiac macrophages showed abnormalities in both M1 and M2 functions post-MI. However, the differences are also obvious. In terms of disease models, we focused on macrophages infiltrating the heart in the acute phase of MI, while Zhang et al. (19) focused on macrophages in white adipose tissue in glucocorticoid-induced obesity, a chronic inflammatory model. In terms of signaling pathways, we studied IFN-γ signaling, while they focused on glucocorticoid signaling. *Klf9* responds to macrophage polarization (Supplemental Figure 7C), which is consistent with a previous study (43).

Recent studies have revealed the role of KLF9 in some heart diseases. The latest research found that KLF9 can regulate mitochondrial energy metabolism and mitophagy by regulating the expression of PGC-1α and mitofusin 2 in cardiomyocytes (26). Therefore, KLF9 deficiency causes the accumulation of dysfunctional mitochondria and accelerates heart failure in response to angiotensin II treatment (26). Correspondingly, we also found that KLF9 deficiency aggravates the deterioration of heart failure. The difference is that we focus on the early repair of MI, while Zhang et al. (26) focused on heart failure caused by angiotensin II. In addition, we found that KLF9 deficiency reduced the level of autophagy in cardiomyocytes (Supplemental Figure 2E), which was better explained by Zhang et al. (26). However, Zhang et al. believed KLF9 knockout would spontaneously result in myocardial hypertrophy and impaired cardiac function, whereas we did not observe this phenomenon, which may be due to differences in mouse strains. Furthermore, we noted that some previous studies reported findings contrary to ours, possibly because the study used KLF9-knockdown rats rather than *Klf9*^–/–^ mice (44) or because the establishment of the MI model might be unstable (45).

We demonstrated that *Klf9* can inhibit the expression of *Stat1* in macrophages, and this regulation can be observed only after stimulation by pro-inflammatory mediators. *Klf9* may inhibit *Stat1* expression by recruiting the transcriptional repressor Sin3A through its Sin3A binding site (46). Our results also show that *Klf9* has a transcriptional activation effect on the promoter of *Stat1* (Figure 9E). We have observed this phenomenon only in vivo but have not been able to mimic the conditions that trigger positive regulation of *Stat1* by *Klf9* in macrophages in vitro. Since the positive regulation of *Stat1* is related to the chemotaxis of macrophages post-MI, we speculate that this positive regulation may occur in myeloid progenitor cells or monocytes, which requires more research to reveal.

Our initial findings indicate that KLF9 protein levels respond to MI (Figure 1B); however, the precise regulatory mechanisms remain unclear. Since *Klf9* mRNA levels in the injured zone post-MI continue to decrease (Supplemental Figure 7D), the increase in protein levels is likely attributable to posttranscriptional regulation. In addition, since cardiomyocytes demonstrate elevated KLF9 protein levels under glucose deprivation, part of the upregulation of KLF9 is contributed by cardiomyocytes, whereas macrophages also expressed higher levels of KLF9 at day 3 post-MI (Supplemental Figure 7E).

In summary, our study found that *Klf9* regulates the response of monocytes/macrophages to IFN-γ signals by regulating the expression and phosphorylation of STAT1 post-MI. Specifically, *Klf9* enhances the chemotaxis of macrophages and inhibits excessive inflammatory response in the early stage of MI, thereby promoting moderate repair post-MI. *Klf9* can regulate the balance between pro-inflammatory and antiinflammatory effects in monocytes/macrophages, and its agonists may serve as potential means for future treatment of post-MI repair.

## Methods

### Sex as a biological variable.

Male mice were used for the study to obviate any confounding effect of variable estrogen effect on MI (47). Moreover, previous studies have found that KLF9 interacts with estrogen receptor and progesterone receptor (15, 48).

### Animals.

*Klf9*^–/–^ mice (C57BL/6JCya-*Klf9^em1^*/Cya) were generated by Cyagen Biosciences, Inc (S-KO-02803). Genotyping primers for *Klf9*^–/–^ were as follows: forward 5′-TGTAGCGTTTGCAGGAAGTCAC-3′; reverse 5′-AAGACCACACCTCTCTCCTAAG-3′. The knockout of *Klf9* gene was validated by genotyping. Male C57BL/6J mice were purchased from Shanghai SLAC Laboratory. Male mice aged 8–12 weeks were used in the experiments. All mice were anesthetized with 2% isoflurane and were euthanized under deep anesthesia with sodium pentobarbital (intraperitoneal injection, 150 mg/kg). MI was performed by permanent ligation of the left anterior descending coronary artery. An electrocardiogram confirmed ischemic ST segment elevation after coronary artery ligation. Sham-operated animals underwent the same procedure without coronary artery ligation. Surgeries were performed blinded to the genotypes.

### BMT.

The recipient mice aged 8–10 weeks were given water supplemented with 0.03% sulfamethoxazole and trimethoprim (5:1) 1 week prior to irradiation. The irradiation was given at a total dose of 8 Gy by an animal x-ray irradiator (Rad Source). The donor bone marrow cells isolated from femur and tibiae were injected intravenously into the tail vein of recipient mice within 6 hours after the irradiation (at a cell number of 5 × 10^6^ per mouse). Recipient mice were housed in a facility with an acidified water supply for 4 more weeks prior to construction of the MI model.

### Flow cytometry.

Flow cytometric analysis was performed following standard methods. Titer experiments are used to determine the concentration of fluorochrome-conjugated antibodies. Hearts were isolated, then washed and digested with Liberase (Roche, 5401127001), to obtain cell suspension. Heart and blood erythrocytes were lysed with 1× Pharm Lyse Lysing Buffer (BD Biosciences, 555899) for 10 minutes at room temperature. The cell suspensions were subsequently blocked with Fc-CD16/CD32 antibody and then stained for 30 minutes in the dark at 4°C with surface staining antibodies. For intracellular labeling, after surface staining, Cytofix/Cytoperm Buffer (BD Biosciences, 555028) was used according to the manufacturer’s instructions. After washing with 1× Perm/Wash buffer (BD Biosciences, 51-9008102), cells were then incubated with antibodies for 30 minutes at 4°C for intracellular labeling. Flow cytometric analysis was performed using a BD LSRFortessa X-20. The antibodies included CD16/CD32 (BD Biosciences, 553142, 2.4G2), CD45 (BD Biosciences, 553080, 30-F11), CD11b (BD Biosciences, 564443, M1/70), LY-6C (BD Biosciences, 563011, AL-21), LY-6G (BioLegend, 127633, 1A8), F4/80 (BD Biosciences, 565411, T45-2342), and CD206 (BD Biosciences, 565250, MR5D3). Data analysis was performed using FlowJo v10.

### Western blotting analysis.

Cells and tissues were homogenized in RIPA Buffer (Cell Signaling Technology [CST], 9806) supplemented with Protease inhibitor (Roche, 4693116001) and Phosphatase inhibitor (Roche, 4906845001), thoroughly lysed, and centrifuged at 12,000 rpm. Proteins were separated by SDS-PAGE and transferred onto PVDF membranes (MilliporeSigma, IPVH00010). The membranes were then blocked with 5% bovine serum albumin (BSA) and incubated with primary antibodies, followed by incubation with horseradish peroxidase–conjugated secondary antibody. The antibodies included HSP90 (CST, 4877S), KLF9 (ABclonal, A7196), LC3 (CST, 4108S), STAT1 (CST, 9172S), p-STAT1 (CST, 9167S), iNOS (Novus Biologicals, Bio-Techne, NB300-605), β-tubulin (CST, 2146S), ARG1 (Servicebio, GB11285), Peroxidase Goat Anti-Mouse IgG (Jackson ImmunoResearch, 115-035-003), and Peroxidase Goat Anti-Rabbit IgG (Jackson ImmunoResearch, 111-035-003). Images were captured using a Western blot imaging system (Amersham Imager 600 or BIO-RAD ChemiDocXRS+) and analyzed using ImageJ software (NIH).

### RT-qPCR.

Total RNA was extracted from heart tissues or BMDMs using TRIzol reagent (Invitrogen,15596018CN) followed by chloroform extraction. mRNA was reverse-transcribed using PrimeScript RT reagent kit (TaKaRa, RR037Q). RT-qPCR was performed on LightCycler 96 Real-Time PCR System (Roche) using the TB Green Premix Ex Taq II Mix (TaKaRa, RR820A) according to the manufacturer’s instructions. Target gene expression was normalized to 18S ribosomal RNA expression and represented as fold-change relative to the control group. All primers are detailed in Supplemental Table 1.

### Immunofluorescence and WGA staining.

Heart tissue was embedded in Tissue-Tek O.C.T. compound (Sakura, 4583) and frozen. We sliced the frozen tissue into 5 μm sections and fixed with 4% paraformaldehyde (PFA). Sections were permeabilized with 0.8% Triton X-100 (MilliporeSigma, T8787-50ML) and blocked with 1% BSA at room temperature. Sections were then incubated with primary antibodies at 4°C overnight. Secondary Alexa Fluor–conjugated antibodies were added to the sections for 1 hour at room temperature. The following secondary antibodies were used: Alexa Fluor 594 Donkey Anti-Goat IgG (yeasen, 34312ES60), Alexa Fluor 488 Donkey Anti-Rabbit IgG (yeasen, 34206ES60), Alexa Fluor 488 Donkey Anti-Rat IgG (yeasen, 34406ES60), Alexa Fluor 594 Donkey Anti-Rabbit IgG (yeasen, 34212ES60), and Cy3 Donkey Anti-Mouse IgG (Jackson ImmunoResearch, 715-165-150). The sections were mounted with DAPI-containing mounting medium (SouthernBiotech, 0100-20). WGA (Invitrogen, W11261) staining was used to assess cellular hypertrophy. The following primary antibodies were used: CD68 (Servicebio, GB113109) 1:100, CD206 (R&D Systems, Bio-Techne, AF2535) 1:200, Sarcomeric Alpha Actinin (Abcam, ab9465) 1:100, vWF (Dako, A0082) 1:200, KLF9 (ABclonal, A7196) 1:100, MAC-2 (Cedarlane, CL8942) 1:500, STAT1 (CST, 9172S) 1:200, and Ly-6B.2 (Cedarlane, CL8993AP). Images were acquired on a Nikon confocal microscope or an Olympus IX83 microscope.

### IHC.

Hearts were fixed overnight in 4% PFA, dehydrated with graded alcohol washes, and embedded in paraffin. Paraffin-embedded hearts were cut into 5 μm sections and were deparaffinized and rehydrated for IHC. Antigen retrieval was performed in citrate buffer, and 3% hydrogen peroxide was used to inactivate endogenous peroxidase. The sections were blocked and incubated with primary antibodies at 4°C overnight. Secondary peroxidase-conjugated antibodies were added to the sections for 1 hour at room temperature. ABC HRP Detection Kit (Novus Biologicals, Bio-Techne, PK-6100-NB) and DAB Substrate Kit (Vector Laboratories, SK-4100) were used to detect color. The following primary antibodies used in IHC: COL1 (Abcam, ab34710) 1:100 and α-SMA (Santa Cruz Biotechnology, sc-32251) 1:50. Images of sections were acquired using a Leica DM6 B microscope.

### H&E, Masson’s trichrome, and Sirius red staining.

The 10 μm–thick paraffin sections were deparaffinized and rehydrated. Tissue sections were stained with H&E or Masson’s trichrome to determine the morphological effects and infarct size according to classical protocols. For Sirius red staining, Sirius Red/Fast Green Collagen Staining Kit (Chondrex, 9046) was used according to the manufacturer’s instructions. Images of sections were acquired using a DM6 B microscope. The mean percentage of the fibrotic area was measured and calculated using the ImageJ software.

### Echocardiography.

Sham and MI mice were anesthetized with 2% isoflurane and placed on a heating pad to maintain the body temperature. Echocardiographic images were obtained at 1 and 4 weeks after the operation using the Vevo 2100 system (VisualSonics) and a 30 MHz linear array transducer. The hearts were viewed from the parasternal short axis, and midventricular M-mode echocardiogram was acquired to determine the EF, FS, LVIDd, LVIDs, heart rate, and intraventricular septum and posterior wall thickness.

### BMDM isolate and culture.

Mice at 8–10 weeks old were euthanized, and femoral and tibial bones were then opened with scissors and flushed with PBS. Bone marrow cells were cultured in DMEM (Gibco, 11965118) with 10% FBS (Gibco, 10099-141), penicillin-streptomycin, and 40 ng/mL recombinant M-CSF (PeproTech, 315-02) at 37°C, 5% CO_2_ for 7 days, to differentiate into BMDMs. BMDMs were then treated with fludarabine (MedChemExpress [MCE], HY-B0069), LPS (Beyotime, ST1470), IFN-γ (MCE, HY-P7071), or IL-13 (R&D Systems, Bio-Techne, 413-ML-025/CF).

### Neonatal rat/mouse cardiomyocyte and fibroblast isolate and culture.

Cardiomyocytes and fibroblasts were isolated from neonatal mice or rats (1 to 3 days). In brief, after dissection, hearts were isolated, washed with cold PBS, cut into small pieces, and digested with 0.25% trypsin at 4°C overnight. The following day, we discarded trypsin and digested tissue pieces with 0.1% type 2 collagenase (Gibco, 17101015) at 37°C 4 or 5 times. We collected the digestion solution, filtered, and centrifuged at 800 rpm for 5 minutes to get cells. After static culture for 90 minutes, the nonadherent cells were transferred to a new culture dish and cultured as cardiomyocytes; the adherent cells were cultured and passaged as fibroblasts.

### Cell line culture.

HeLa (human cervical carcinoma), HEK293T (human embryonic kidney cell), and RAW264.7 (murine macrophage) were purchased from Shanghai Cellbank (Shanghai, China). Cells were cultured in DMEM (Gibco, 11965118) with 10% FBS (Gibco, 10099-141), and penicillin-streptomycin at 37°C, 5% CO_2_.

### scRNA-Seq and data analyses.

LV tissues from WT and *Klf9*^–/–^ hearts were isolated 72 hours post-MI, minced, washed in ice-cold RPMI 1640 (Gibco, 11875093), and digested using Multi-tissue dissociation kit 2 (Miltenyi Biotec, 130-110-203) according to the manufacturer’s protocol. Digested cells were filtered and centrifuged at 600*g* to obtain a single-cell suspension. Cell debris and dead cells were removed using Debris Removal Solution (Miltenyi Biotec, 130-109-398). Live cells were washed in RPMI 1640 and then resuspended at 1 × 10^6^ cells/mL in 1× PBS and 0.04% BSA. Each sample was pooled from LV tissues of 2 mice.

Cells were loaded into Chromium microfluidic chips and barcoded within a Chromium Controller (10x Genomics). The single-cell transcriptome was reverse-transcribed into a cDNA library that contained 10x Genomics cell barcodes and unique molecular identifiers constructed with reagents from the Chromium Next GEM Single Cell 5’ Library & Gel Bead Kit v1.1 (10x Genomics,1000165) according to the manufacturer’s protocol. cDNA libraries were sequenced in PE150 mode on the NovaSeq platform (Illumina). The 10x Genomics Cell Ranger (v3.1.0) was used to align the raw data to the mouse reference transcriptome (mm10) and generate gene-cell count matrices.

After sequencing data processing, initial quality control and clustering were performed using Seurat (v5.0.3) pipeline in R (v4.3.3). Briefly, for each dataset, we filtered out potentially low-quality cells using the quality control parameters of nFeature_RNA per cell > 200 and < 5,000, and <15% of mitochondrial genes, and filtered out low-quality genes expressed in <3 cells. The trimmed expression count matrix was log-transformed for downstream processing using the function NormalizeData. Then, the most variable genes were selected using the function FindVariableGenes with default parameters and were used for principal component analysis. A total of 20 principal components were used for RunUMAP and FindNeighbors, and cluster selection was performed using a resolution of 0.6. The FindAllMarkers function in the Seurat package was used with parameters test.use=wilcox, min.pct=0.25, logfc.threshold = 0.2 to obtain the DEGs between specific cell clusters. The cell types were annotated by using SingleR (v2.4.1) based on marker genes and matched to canonical markers. DEGs between WT and *Klf9*^–/–^ groups in different clusters were statistically determined using a Wilcoxon’s rank-sum test with a *P* value threshold of 0.05 and a log2(FC) > 0.25.

GO enrichment analysis were performed by using ClusterProfiler (v4.11.1) with *P* < 0.05 and log2(FC) > 0.5. For pseudotime trajectory analysis, monocytes/macrophages and fibroblasts were subset from the single-cell dataset and run through a similar analysis procedure; then, Monocle3 (v1.2.9) was used to study pseudotime trajectory of cells.

### Plasma cTnI and LDH assay.

Mice were anesthetized, blood was drawn and anticoagulated with 2 mg/mL EDTA, and plasma was obtained after centrifugation at 3,000 rpm for 10 minutes. The plasma level of cTnI in MI mice was measured using an ELISA kit (Life Diagnostics, CTNI-1-HSP), and the plasma level of LDH was analyzed using a Lactate Dehydrogenase Assay Kit (Beyotime, P0395S).

### ChIP.

FLAG-KLF9 stable overexpression was achieved using lentiviral infection. The lentiviral vector pCDH-EF1-3xFlag-MCS-T2A-puro encoding mouse KLF9 (Lenti-KLF9) was constructed, packaged, and purified. RAW264.7 cells were infected with Lenti-KLF9 lentivirus,and FLAG-KLF9 was expressed in the cells by lentiviral transduction. At 48 hours after infection, cells with puromycin resistance were selected by treatment with puromycin for 7 days. ChIP assay was performed on RAW264.7 cells stably overexpressing FLAG-KLF9 using SimpleChIP Enzymatic Chromatin IP Kit (CST, 9003), according to the manufacturer’s instructions. Briefly, RAW264.7 cells were cross-linked with 1% formaldehyde, neutralized with glycine, lysed, and centrifuged at 2,000*g* to collect cell nuclei. Chromosomal DNA was digested to a length of approximately 150–900 bp by Micrococcal Nuclease (CST, 10011), and the cell nuclei were washed and sonicated. Digested chromatin was immunoprecipitated using anti-FLAG antibody (MilliporeSigma, A8592) or IgG (CST, 2729). Then, Protein G Magnetic Beads were added to the ChIP reaction mixture and incubated. After washing, chromatin was eluted from the Protein G beads to reverse the cross-links. Proteinase K digests were performed and DNA was purified using spin columns and quantified by RT-qPCR and PCR using primers listed in Supplemental Table 2.

### Luciferase reporter assay.

The promoter element of mouse *Stat1* gene (mouse GRCm39) was cloned by PCR (P1: GRCm39 chromosome 1: 52152400-52153401, P2: GRCm39 chromosome 1: 52157800-52159182). The promoter fragments were separately inserted into PGL3-basic vector to obtain PGL3-*Stat1* vector (PGL3-promoter1 and PGL3-promoter2). Subsequently, the overexpression vector PCDNA3.1-KLF9 (KLF9) encoding mouse KLF9 was constructed. For luciferase assay, HEK293T cells were seeded and transiently transfected with PGL3-basic, promoter1+ PCDNA3.1, promoter1+ KLF9, promoter2+ PCDNA3.1, promoter2+ KLF9, using Lipofectamine 2000 (Invitrogen, 11668030). About 72 hours after the transfection, cells were lysed, and we measured luciferase activity with the Luciferase Reporter Gene Assay Kit (Beyotime, RG010S) according to the manufacturer’s instructions. Luciferase activities were determined using a multimode microplate detection system (SpectraMax i3).

### Graphical abstract.

The graphical abstract was created using BioRender with Publication and Licensing Rights.

### Statistics.

All experiments were analyzed using GraphPad Prism 8. Quantitative data are shown as means ± SEM. Statistical differences between the 2 groups were analyzed using unpaired 2-tailed Student’s *t* test. One-way or 2-way ANOVA with Bonferroni’s correction was used for multiple comparisons. The Kaplan-Meier method with a log-rank test was used for survival analysis. *P* < 0.05 was considered statistically significant.

### Study approval.

All animal experiments were approved by the Animal Research Committee of Shanghai Tenth People’s Hospital and Tongji University Animal Research Committee.

### Data availability.

Mouse single-cell sequencing data used in this article have been deposited in National Center for Biotechnology Information Gene Expression Omnibus (GSE272924). Human single-cell sequencing data are published and are available at cellxgene (22). Other datasets are available in the Supporting Data Values XLS file.

## Author contributions

SX performed most of the experiments and data analysis. HL, YX, JH, and NL helped with the experiments. WC and WY wrote and revised the manuscript. FL and WY designed the study.

## Supplementary Material

Supplemental data

Unedited blot and gel images

Supporting data values

## Version 1 04/08/2025

In-Press Preview

## Version 2 05/08/2025

Electronic publication
