# Supplementary material for: Klf9 promotes the repair of myocardial infarction by regulating macrophage recruitment and polarization
Source: JCI Insight. 2025 Apr 8;10(9):e187072. doi: 10.1172/jci.insight.187072 (PMC12128982; doi:10.1172/jci.insight.187072)
Supplement: Supplemental data [file jciinsight-10-187072-s039.pdf]

Klf9 promotes the repair of myocardial infarction by regulating macrophage recruitment and polarization

Authors:

Sheng Xu[1], Hao Li[1], Jun Han[1], Yawei Xu[1], Niannian Li[2], Wenliang Che[1*], Feng Liu[2*], Wenhui Yue[1*]

Affiliations:

[1] Department of Cardiology, Shanghai Tenth People's Hospital, Tongji University School of Medicine, China

[2] Department of Otolaryngology Head and Neck Surgery, Shanghai Key Laboratory of Sleep Disordered Breathing, Shanghai Jiao Tong University School of Medicine Affiliated Sixth People's Hospital, China

[*] corresponding author:

Wenhui Yue, email: yuewenhui@tongji.edu.cn

Feng Liu, email: liufeng@sibs.ac.cn

Wenliang Che, email: chewenliang@tongji.edu.cn

Supplemental Figure 1

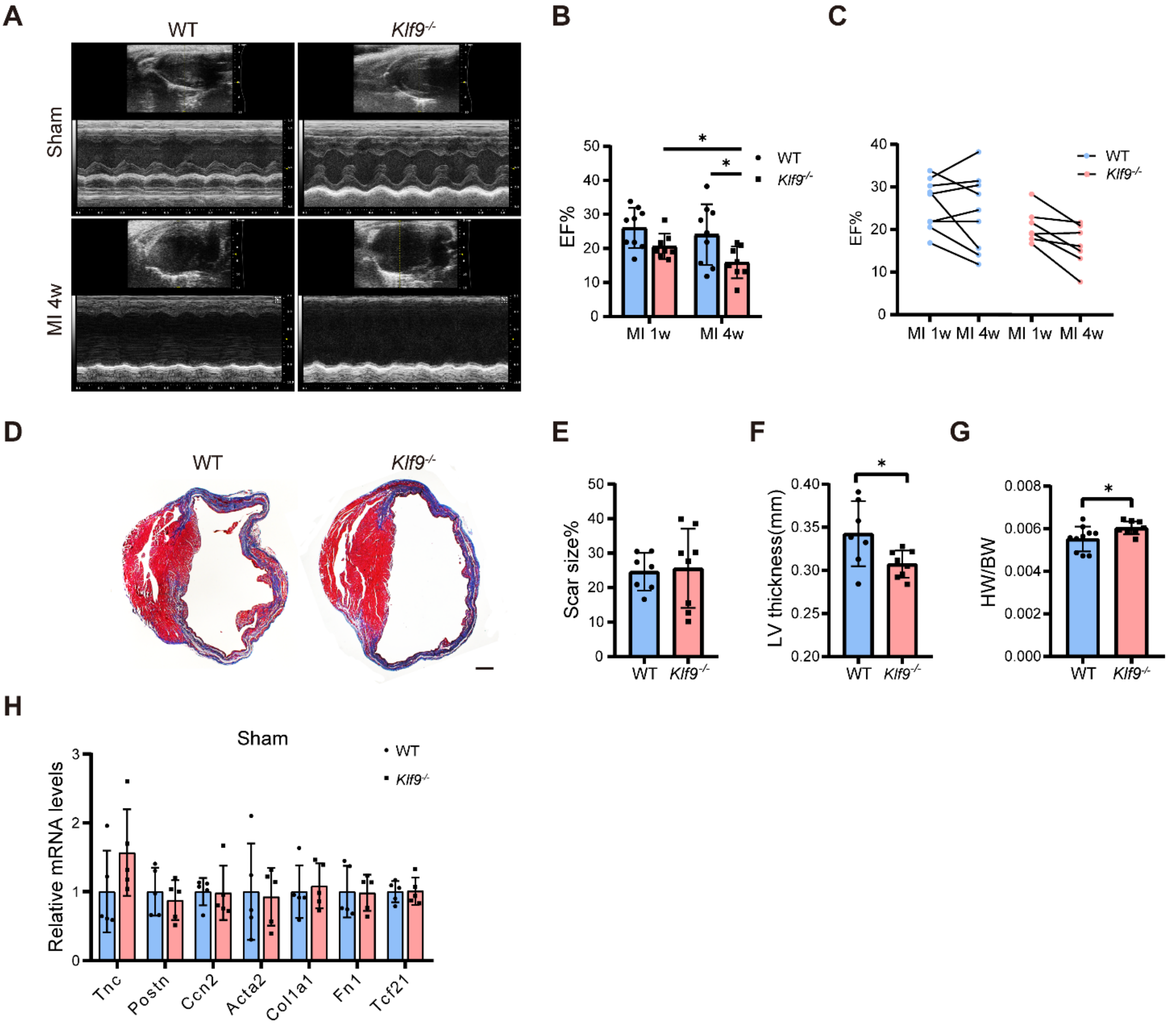

Supplemental Figure 1. KLF9 deficiency deteriorated the heart failure post-MI
(**A**) Representative M-mode images of WT and *Klf9*$^{-/-}$ mice 4 weeks post-MI. (**B-C**) The measurement of Left ventricular ejection fraction (EF) and the change of EF% between 1 week and 4 weeks post-MI (n =8-9). (**D**) Representative Masson's trichrome staining and (**E-F**) quantitative data of scar size% and LV thickness at 4 weeks post-MI (n =7-8, Scale bar=0.5mm). (**G**) Ratio of heart weight to body weight (HW/BW) in WT and *Klf9*$^{-/-}$ groups 4 weeks post-MI (n =9-10). (**H**) RT-qPCR of myofibroblast activation marker genes in cardiac tissues from Sham group (n = 5). Each point represents a mouse sample and all data are expressed as means ± S.E.M. Unpaired two-tailed Student's t-test (**E-H**), paired two-way ANOVA (**B**) was used for statistical analyses. *p<0.05.

Supplemental Figure 2

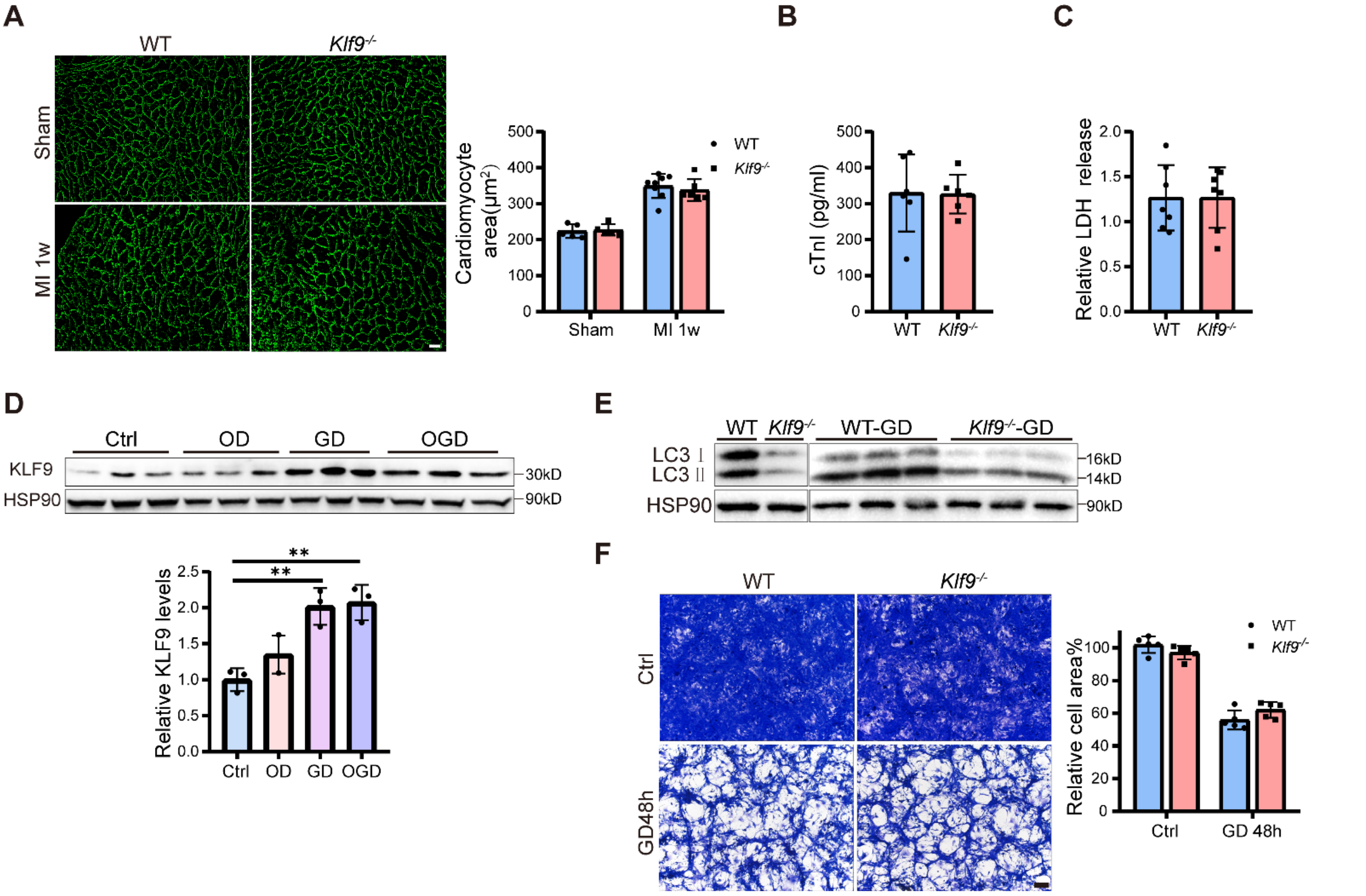

Supplemental Figure 2. KLF9 deficiency does not increase cardiomyocyte death

(**A**) Representative images of WGA staining and cardiomyocyte area measurement at 1-week post-MI. (Sham n=5, MI n=6-8, Scale bars=20μm). (**B**) ELISA results of mouse plasma cardiac troponins I (cTnI) levels at day 3 post-MI (n=6). (**C**) Relative lactate dehydrogenase (LHD) levels in plasma at day 3 post-MI (n=7). (**D**) Expression of KLF9 in neonatal rat cardiomyocytes treated with oxygen deprivation (OD), glucose-serum deprivation (GD), and oxygen-glucose deprivation (OGD) for 12 hours, and the quantitative statistics (n=3). (**E**) Expression of LC3 in neonatal mouse cardiomyocytes after 12h of GD treatment. (**F**) Representative images and quantitative statistics of crystal violet staining in neonatal mouse cardiomyocytes after 48 hours of GD. (n=5, Scale bars=100μm). Each point represents a mouse sample (**A-C**) or a cell sample (**D, F**) and all data are expressed as means ± S.E.M. Unpaired two-tailed Student's t-test (**A-C, F**), one-way ANOVA (**D**) was used for statistical analyses. *p<0.05, **p<0.01.

Supplemental Figure 3

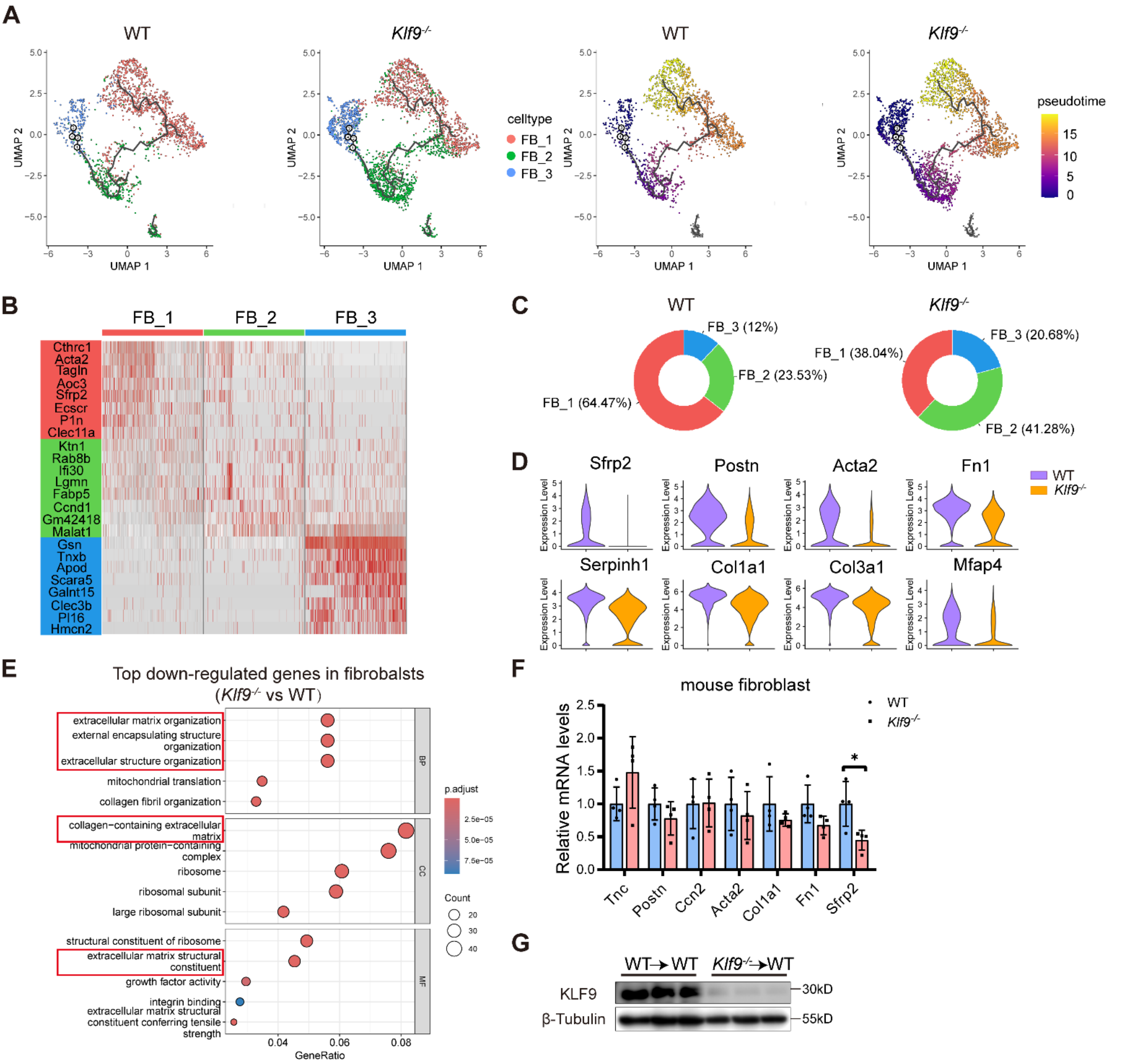

Supplemental Figure 3. Abnormal function of KLF9 deficient macrophages inhibits fibroblast activation

(**A**) Pseudo-time trajectory analysis in fibroblast subsets, cells were colored by pseudo-time or subcluster labels. 1925 WT fibroblasts and 2781 *Klf9*$^{-/-}$ fibroblasts are shown in the UMAP. (**B**) Expression heatmap of marker genes in different fibroblast subclusters. (**C**) The proportion of each subcluster in the fibroblasts in the WT and *Klf9*$^{-/-}$ groups. (**D**) Violin plot of some extracellular matrix pathway genes downregulated in *Klf9*$^{-/-}$ group in fibroblasts. (**E**) GO enrichment analysis of the most significantly downregulated genes in fibroblasts (differential gene KO vs WT: log2(FC)<-0.5). (**F**) Expression of fibroblast activation-related genes in neonatal mouse cardiac fibroblasts(n=4). (**G**) Expression of KLF9 in blood nucleated cells of mice 4 weeks after bone marrow transplantation. Each point represents a cell sample and unpaired two-tailed Student's t-test (**F**) was used for statistical analyses. *p<0.05.

Supplemental Figure 4

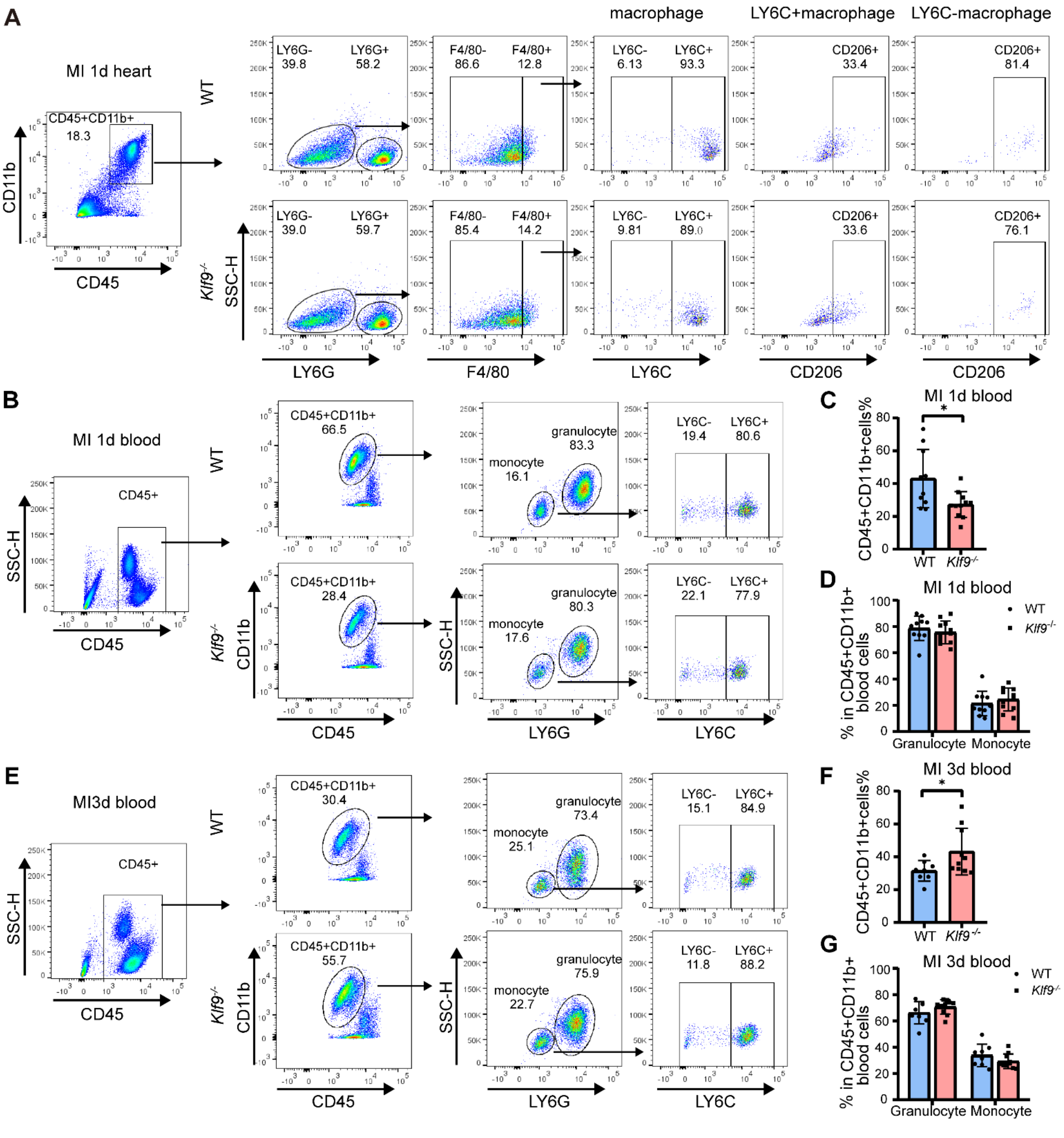

Supplemental Figure 4. KLF9 deficiency reduces myeloid cells in the early stages of MI.
(**A**) Flow cytometry gating strategy and representative flow cytometric analysis of macrophages, granulocytes, and monocytes in WT and *Klf9*$^{-/-}$ heart 1-day post-MI, 20000 CD45+ cells were collected from each mouse sample. (**B, E**) Flow cytometry gating strategy and representative flow cytometric analysis of CD45+CD11b+myeloid cells, granulocytes, and monocytes in WT and *Klf9*$^{-/-}$ blood 1 day (**B**) and 3 days (**E**) post-MI, 20000 CD45+ cells were collected from each mouse sample. (**C, F**) quantification of the proportion of CD45+CD11b+ myeloid blood cells to CD45+ immune cells in the blood by flow cytometry 1 day (**C**) and 3 days (**F**) post-MI (n= 8-11). (**D, G**) quantification of monocytes and LY6G+ granulocytes in the blood by flow cytometry 1 day (**D**) and 3 days (**G**) post-MI (n= 8-11). Each point represents a mouse sample and all data are expressed as means ± S.E.M. Unpaired two-tailed Student's t-test (**C-D, F-G**) was used for statistical analyses. *p<0.05.

Supplemental Figure 5

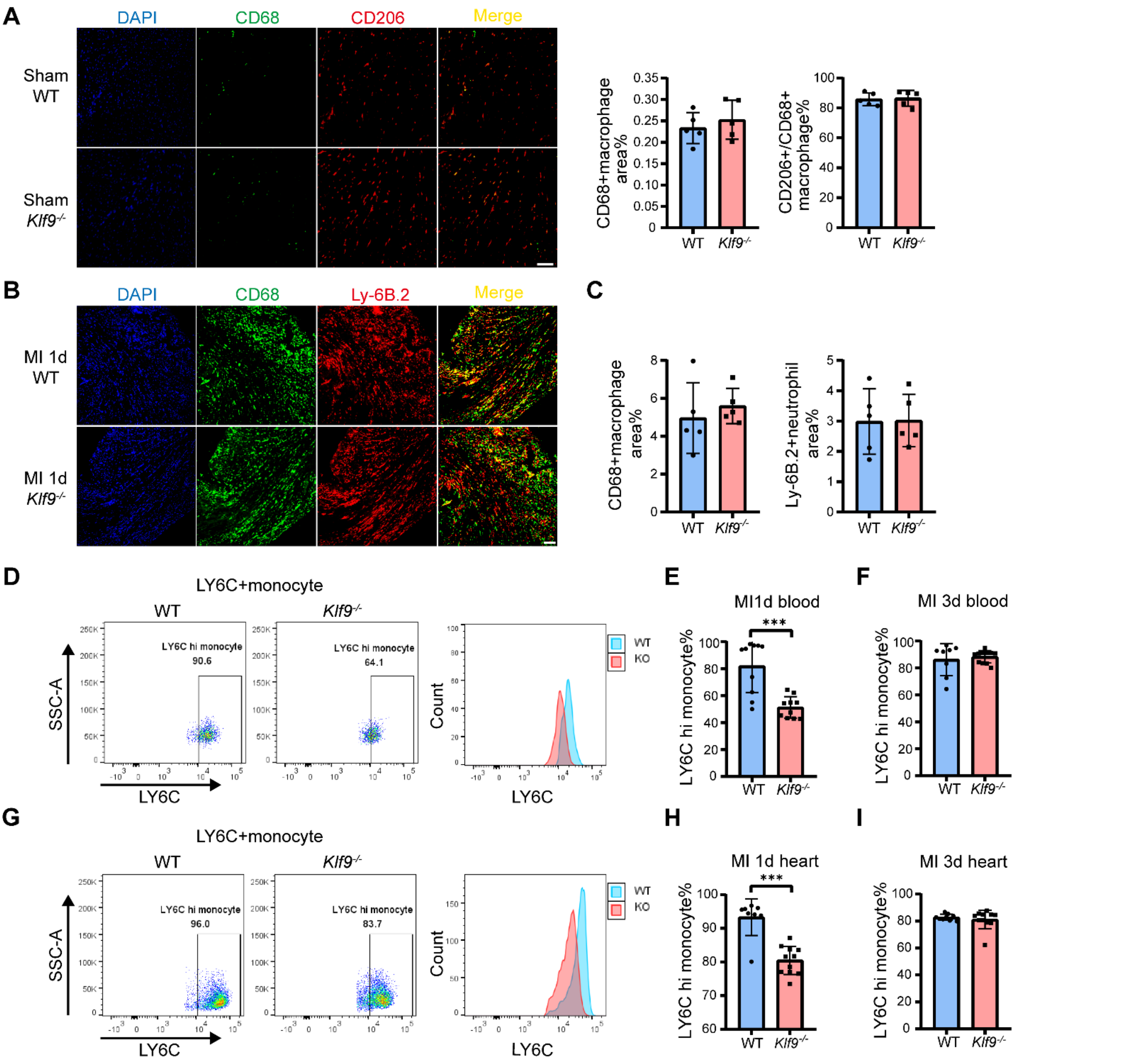

Supplemental Figure 5. Effect of KLF9 deficiency on neutrophils and monocytes post-MI

(**A**) Quantification of CD68+macrophage and CD68+CD206+M2 macrophages after Sham operation. (n =5, Scale bars=100μm). (**B-C**) Representative immunofluorescent images and quantification of CD68+macrophage and Ly-6B.2+neutrophils at day 1 post-MI. (n =5, Scale bars=100μm). (**D-F**) Representative flow cytometric analysis and quantification of the proportion of LY6C high monocytes to LY6C+ monocytes in the blood at day 1 (**D-E**) and day 3 (**F**) post-MI (n=8-11). (**G-I**) Representative flow cytometric analysis and quantification of the proportion of LY6C high monocytes to LY6C+ monocytes in the heart at day 1 (**G-H**) and day 3 (**I**) post-MI (n= 8-11)). Each point represents a mouse sample and all data are expressed as means ± S.E.M. Unpaired two-tailed Student's t-test (**A, C, E-F, H-I**) was used for statistical analyses. *p<0.05, **p<0.01, ***p<0.001

Supplemental Figure 6

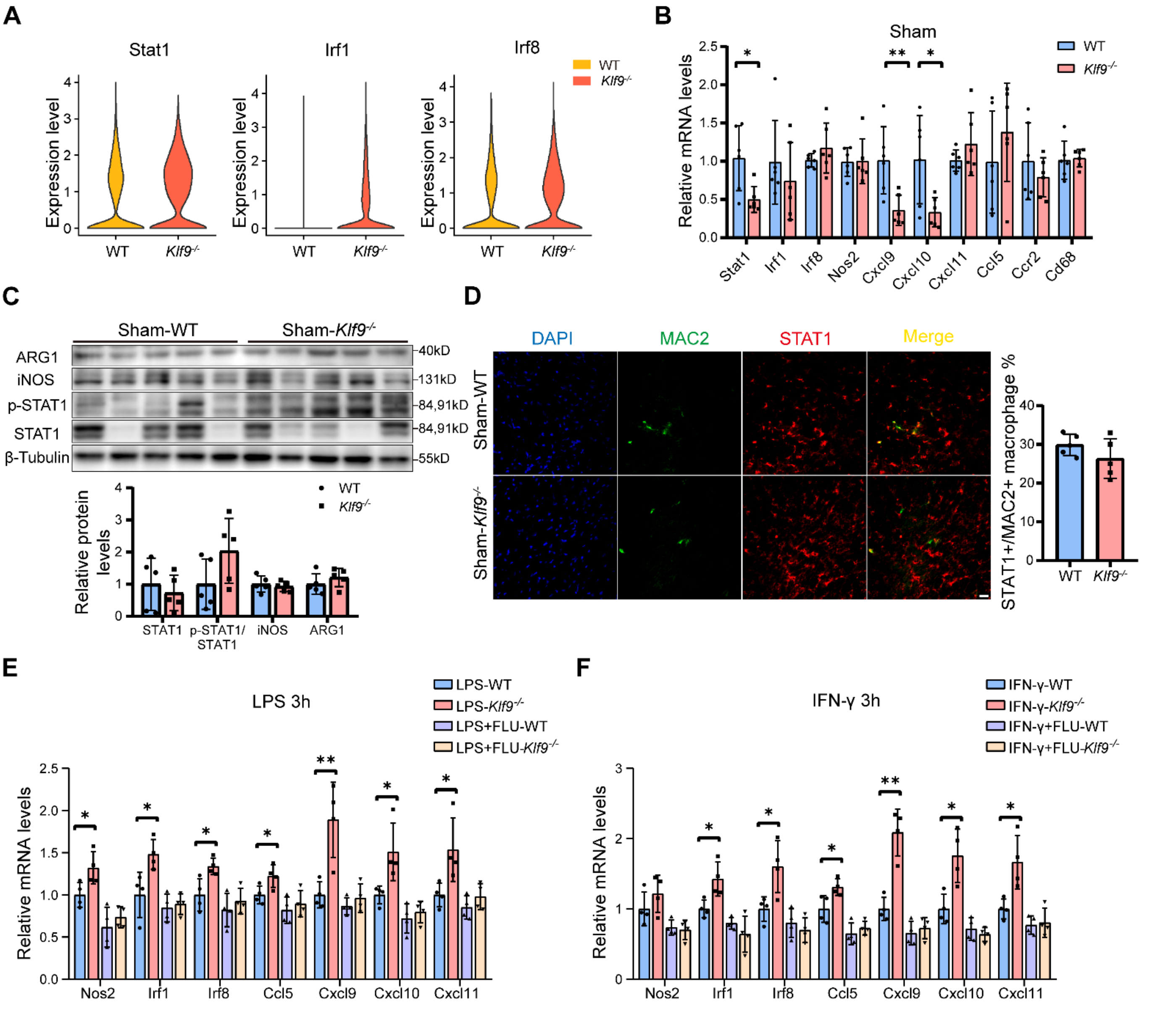

Supplemental Figure 6. Expression of STAT1 in macrophages and Sham group

(**A**) Violin plots of *Stat1* and its related genes expression in macrophages from scRNA-seq data. (**B**) Relative mRNA expression levels of STAT1 pathway-related genes and *Ccr2*, *Cd68* in the hearts after Sham operation (n=6). (**C**) STAT1, p-STAT1, iNOS and ARG1 expression and Quantification in the hearts after Sham operation (n=5). (**D**) Immunofluorescent images and quantification of percentage of STAT1+MAC2+macrophages to total macrophages area in the hearts after Sham operation (n=5, Scale bars=20μm). (**E-F**)BMDMs pretreated with DMSO or 50 μ M fludarabine for 6 h, and then stimulated with LPS (**E**) or IFN-γ (**F**) for 3h, followed by analysis of *Stat1* target genes expression by qRT–PCR. (n=4, LPS 50ng/ml, IFN-γ 50ng/ml). Each point represents a mouse sample (**B-D**) or a cell sample (**E-F**), and all data are expressed as means ± S.E.M. Unpaired two-tailed Student's t-test (**B-F**) was used for statistical analyses. *p<0.05, **p<0.01.

Supplemental Figure 7

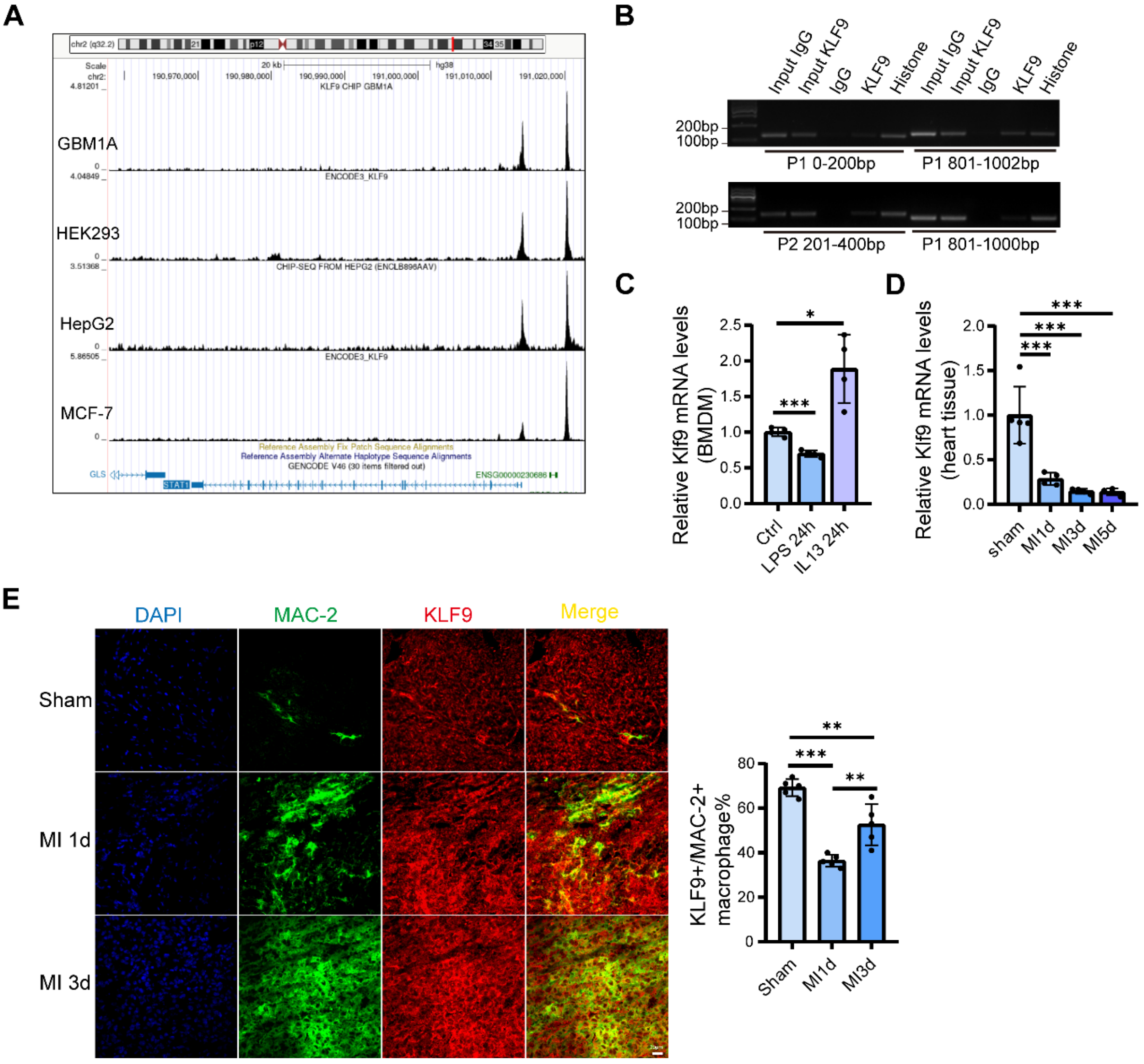

Supplemental Figure 7. KLF9 regulates macrophage STAT1 by binding to the *Stat1* promoter.
(**A**) The binding of KLF9 to the STAT1 gene promoter in several human cells from the ChIP-seq public database (Cistrome DB). (**B**) ChIP was performed against KLF9 on FLAG-KLF9 overexpressing RAW264.7 cells followed by PCR. (**C**) mRNA expression of *Klf9* in BMDMs after LPS and IL-13 stimulation (n=4). (**D**) *Klf9* mRNA levels in the injured zone of heart after Sham and MI at different time points (n=4-5). (**E**) Immunofluorescent images and quantification of percentage of KLF9+MAC2+macrophages to total macrophages area in the hearts after Sham and MI operation (n=5, Scale bars=20μm). Each point represents a mouse sample (**D-E**) or a cell sample (**C**) and all data are expressed as means ± S.E.M. Unpaired two-tailed Student's t-test (**C**), one-way ANOVA (**D-E**) was used for statistical analyses. *p<0.05, **p<0.01, ***p<0.001.

Supplemental Table 1 qPCR primers

Gene	Forward (5'->3')	Reverse (5'->3')
18S	GCAATTATTCCCCATGAACG	GGCCTCACTAAACCATCCAA
Irf1	GGGCATCTTTCGCTTCGTG	GCCCTGGGATTTGGTTGGAA
Irf8	CTCTAGGATACAGGCTCTGATTGC	TTCCCTTTAAAAACTGCCCAGG
Stat1	GCGCTGCTTGGCTCTCTTA	CCAGGAACTTGGAGTCCAGC
Csf1r	TGGCGAGGGTTCATTATCCG	CCAGCTTGCTAGGCTCCAAT
Arg1	CTCCAAGCCAAAGTCCTTAGAG	AGGAGCTGTCATTAGGGACATC
Cxcl9	AACGGAGATCAAACCTGCCT	CTTCCTTGAACGACGACGAC
Cxcl10	TGCCGTCATTTTCTGCCTCA	AGGCTCGCAGGGATGATTTC
Cxcl11	AACGACAAAGGTGCCTGGAC	TCCAAGACAGCAGAGGGTCA
Ccl5	CACCATATGGCTCGGACACC	TCTGGGTTGGCACACACTTG
Nos2	CATCAACCAGTATTATGGCTC	TTTCCTTTGTTACAGCTTCC
Cd68	TGTCTGATCTTGCTAGGACCG	GAGAGTAACGGCCTTTTTGTGA
Ccr2	GTTCAGCTGCCTGCAAAGAC	ATGCCGTGGATGAACTGAGG
Tnf	GCCTCTTCTCATTCCTGCTTG	CTGATGAGAGGGAGGCCATT
Il1b	TGCCACCTTTTGACAGTGATG	TGATGTGCTGCTGCGAGATT
Tnc	CTCCCAGCATCCGTACCAAA	GATGCCGTCCAGGAAACTGT
Ccn2	AGAACTGTGTACGGAGCGTG	GTGCACCATCTTTGGCAGTG
Col1a1	GCTCCTCTTAGGGGCCACT	CCACGTCTCACCATTGGGG
Postn	TGGTATCAAGGTGCTATCTGCG	AATGCCCAGCGTGCCATAA
Acta2	GTCCCAGACATCAGGGAGTAA	TCGGATACTTCAGCGTCAGGA
Tcf21	CTGGCCAACGACAAGTACGA	TGTAGTTCCACACAAGCGGT
Fn1	AGGCAATGGACGCATCAC	TTCCTCGGTTGTCCTTCTTG

Supplemental Table 2 CHIP-qPCR primers

Gene	Forward (5'->3')	Reverse (5'->3')
Stat1-p1 0-200bp	ACCAAGACTGGCATCGAGTT	AAAACCAGTTTGGCCCCTTG
Stat1-p1 201-400bp	CCCATTTAGGCCTTGAGCAAA	CATTTTTCTCGTGGCCCTTCC
Stat1-p1 401-600bp	TTTCCATCGTCCTGAGTCTGC	TCGAAAAGCGAAAGCAATGCC
Stat1-p1 601-800bp	CGGCTGAGTTCCCAGAAAGC	CAGGCTTTGCTTTAGGACCC
Stat1-p1 801-1002bp	GGTGTGCATGCGATCCAAGA	TGCTGACAAAGACACTGGAAAA
Stat1-p2 0-200bp	TTGGAAGAAAGCAGTGGGTGT	CCCGTTGTGCCTTTGATGAC
Stat1-p2 201-400bp	GCGGGACAAAAGTTTCGGT	TCAACCAAGCCTGCAAGAAG
Stat1-p2 401-600bp	GCAAATCCAAGCCCTGTCCT	ACGCTACTAAAGCCAGGGAG
Stat1-p2 601-800bp	TGTAAACACAACCCCCTGTCTG	ATTGGCCGGCTGTTCCAT
Stat1-p2 801-1000bp	AGCGCCGAGTCTGTCAAAG	TCGAGTCTGGGCAAATCTCTC
Stat1-p2 1001-1200bp	GGATCGCTTGCCCAACTCTT	CCGGGAGAAATGTCTATTGCAC
Stat1-p2 1201-1383bp	GCAGTCGTTTCAGCTCTGCT	CAAGCAATCACCACACACAGC
