# Supplementary material for: Klf9 promotes the repair of myocardial infarction by regulating macrophage recruitment and polarization
Source: JCI Insight. 2025 Apr 8;10(9):e187072. doi: 10.1172/jci.insight.187072 (PMC12128982; doi:10.1172/jci.insight.187072)

All lanes were used for the figures

Full unedited blot for Figure 1 B

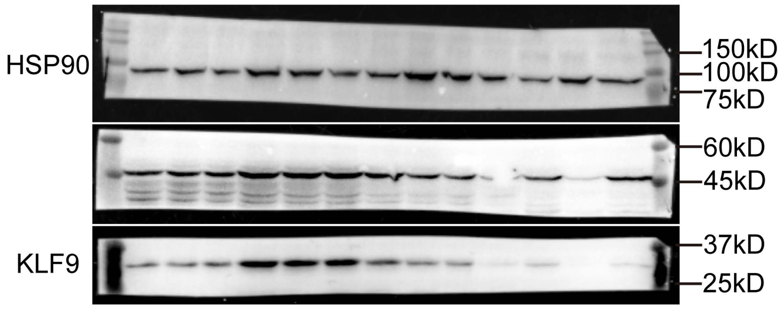

Full unedited blot for Supplemental Figure 2 D

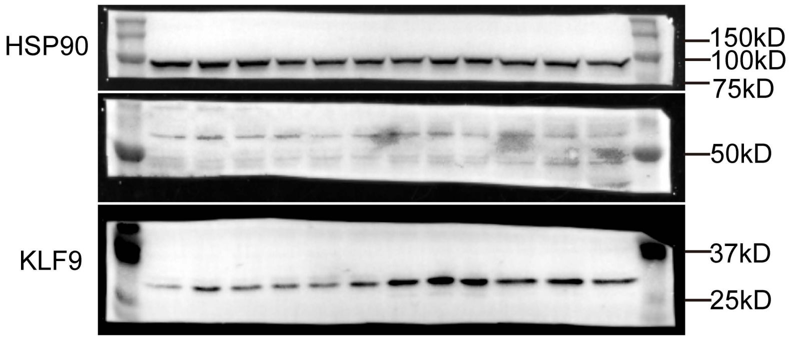

Full unedited blot for Supplemental Figure 2 E

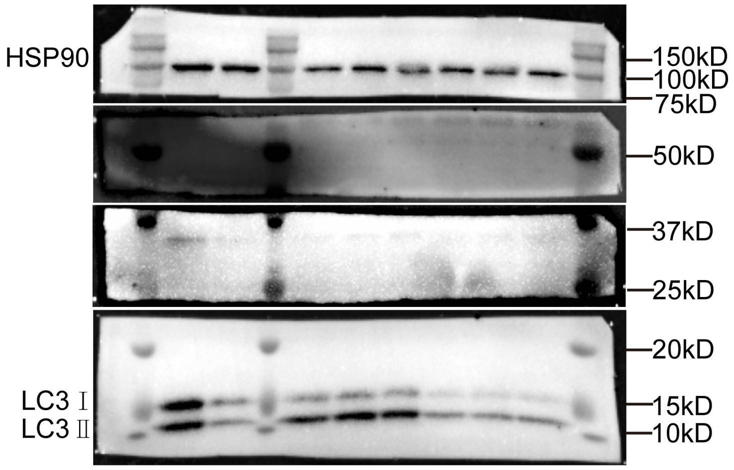

Full unedited blot for Supplemental Figure 3 G

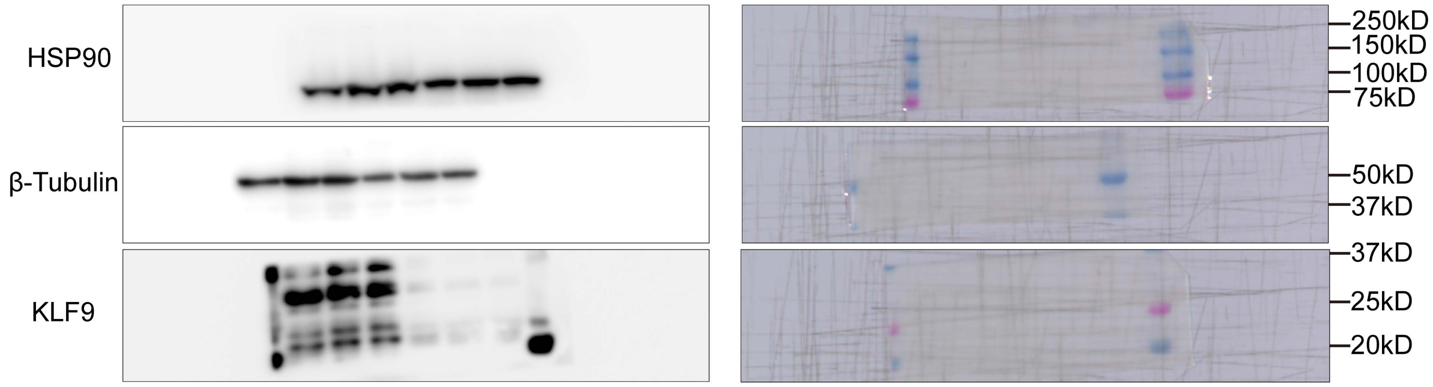

Full unedited blot for Supplemental Figure 6 C

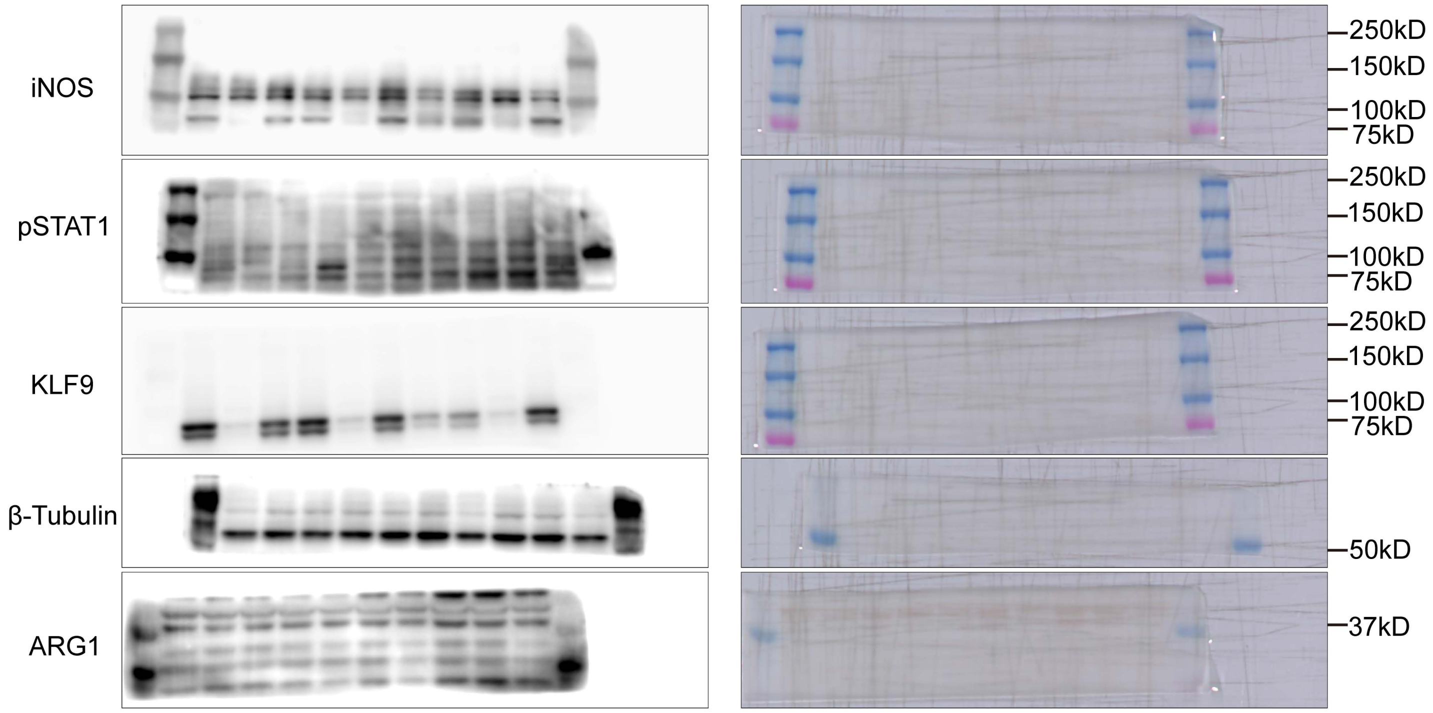

All lanes were used for the figures

Full unedited blot for Figure 7 E

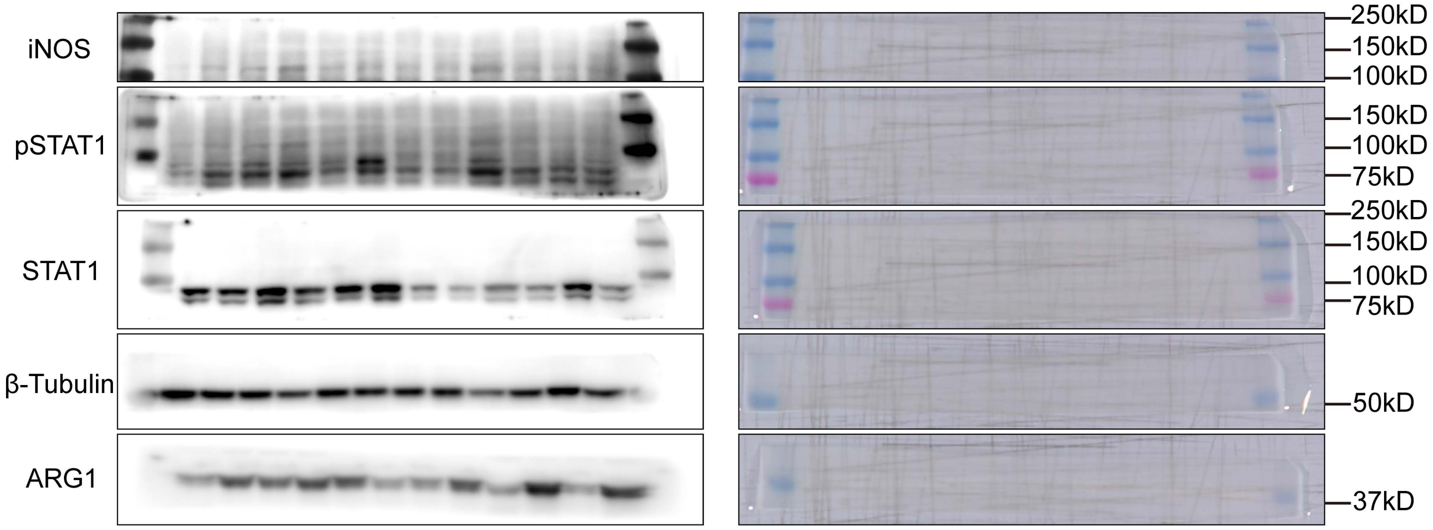

Full unedited blot for Figure 7 F

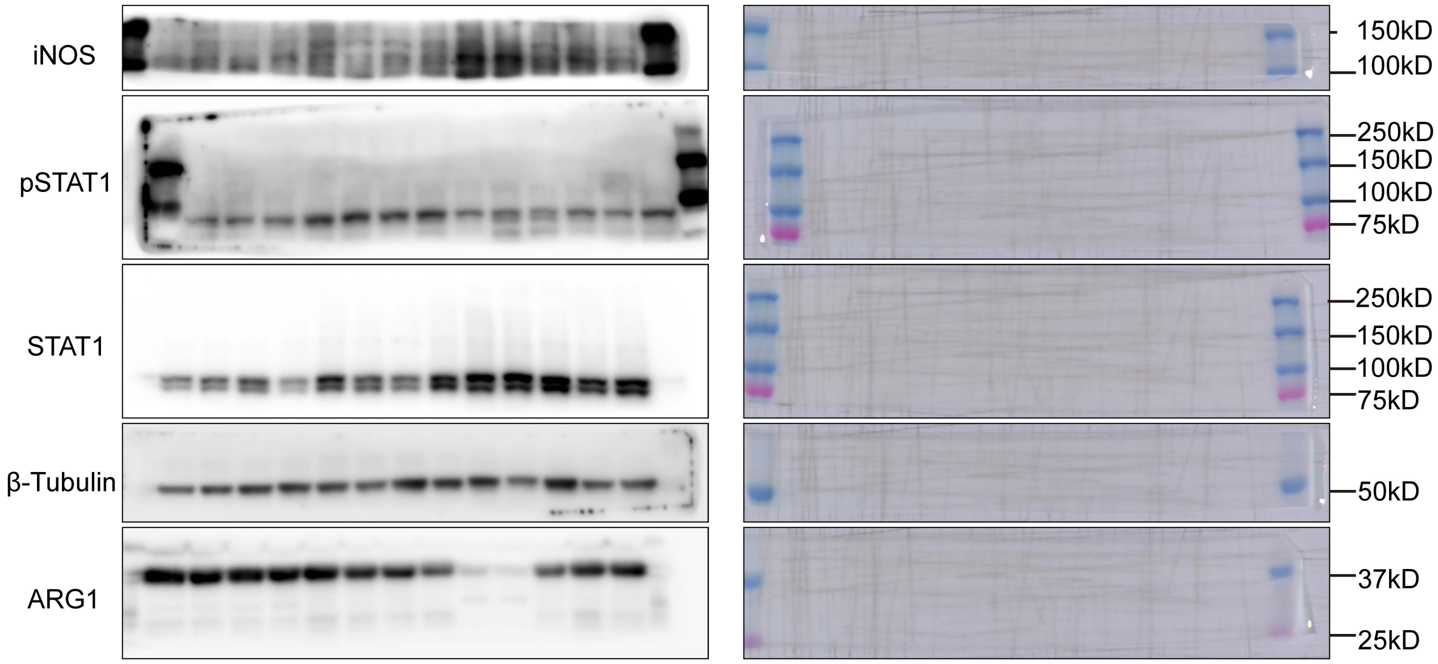

Full unedited blot for Figure 8 C

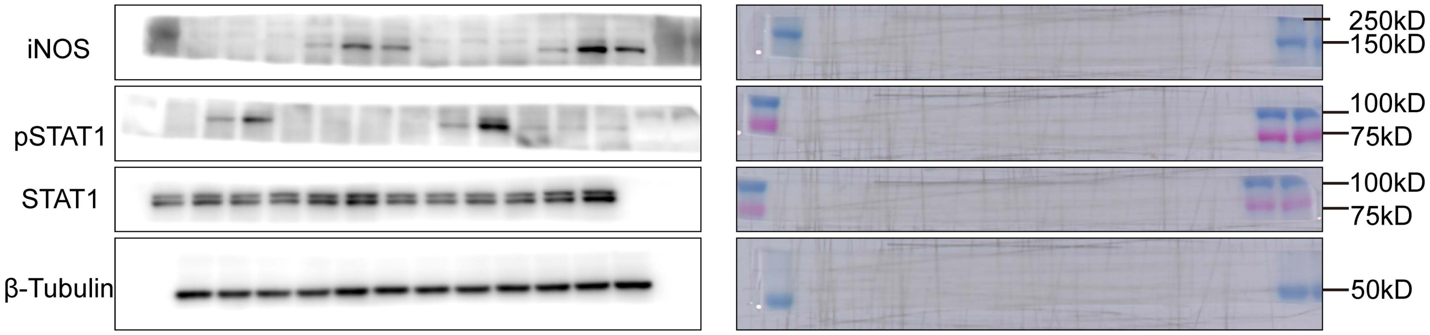

All lanes were used for the figures

Full unedited blot for Figure 9 A

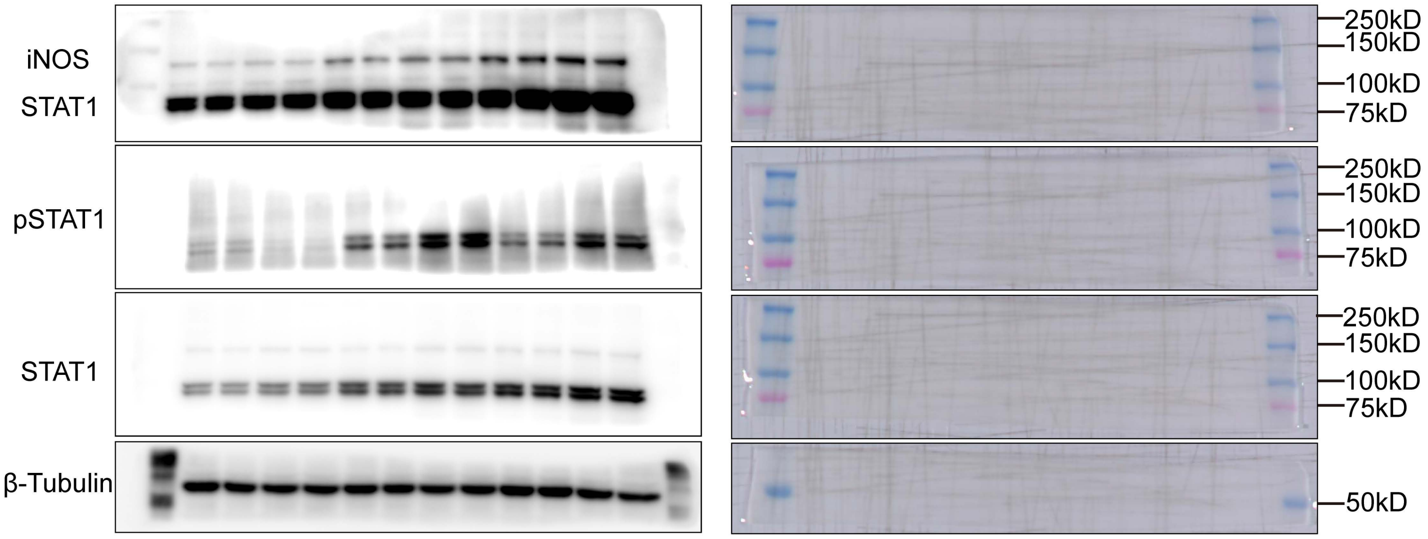

Full unedited blot for Figure 9 B

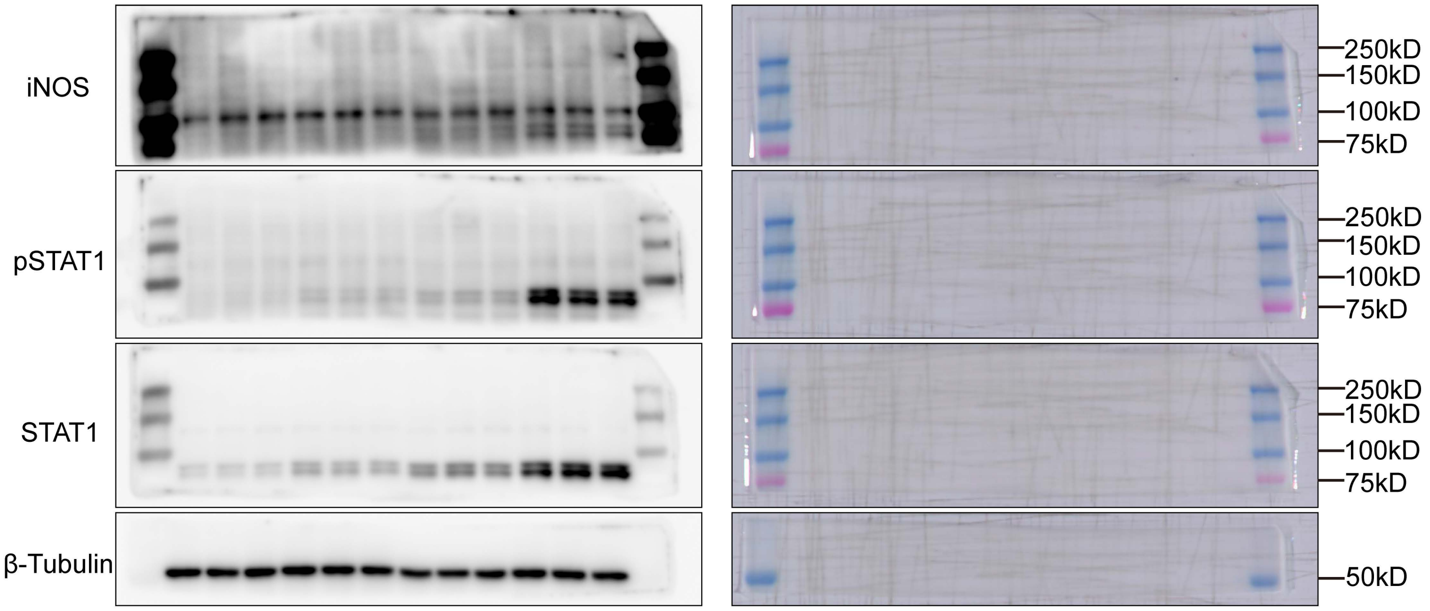

Full unedited blot for Figure 9 C

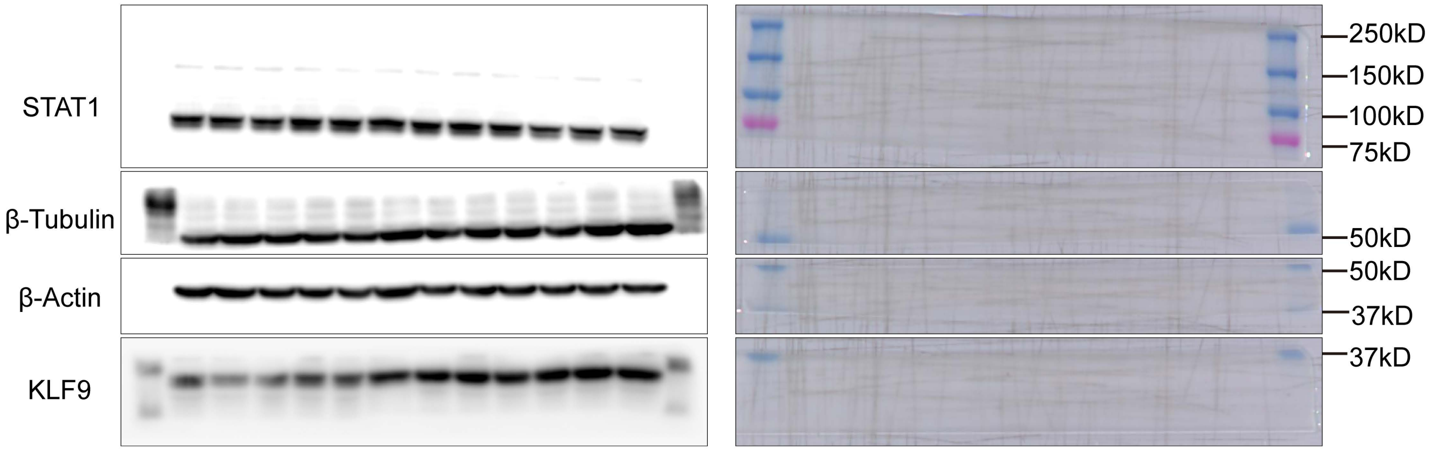

Supplement: Unedited blot and gel images [file jciinsight-10-187072-s040.pdf]
